# Structural basis of TRPV1 modulation by endogenous bioactive lipids

**DOI:** 10.1038/s41594-024-01299-2

**Published:** 2024-05-02

**Authors:** William R. Arnold, Adamo Mancino, Frank R. Moss, Adam Frost, David Julius, Yifan Cheng

**Affiliations:** 1 Department of Biochemistry and Biophysics, University of California San Francisco, San Francisco, CA USA; 2 Biophysics Graduate Program, University of California San Francisco, San Francisco, CA USA; 3 Department of Physiology, University of California San Francisco, San Francisco, CA USA; 4 Howard Hughes Medical Institute, University of California San Francisco, San Francisco, CA USA; 5 Present Address: Altos Labs, Redwood City, CA USA; 6 Present Address: Chan Zuckerberg Biohub, San Francisco, CA USA

**Keywords:** Cryoelectron microscopy, Transient receptor potential channels

## Abstract

TRP ion channels are modulated by phosphoinositide lipids, but the underlying structural mechanisms remain unclear. The capsaicin- and heat-activated receptor, TRPV1, has served as a model for deciphering lipid modulation, which is relevant to understanding how pro-algesic agents enhance channel activity in the setting of inflammatory pain. Identification of a pocket within the TRPV1 transmembrane core has provided initial clues as to how phosphoinositide lipids bind to and regulate the channel. Here we show that this regulatory pocket in rat TRPV1 can accommodate diverse lipid species, including the inflammatory lipid lysophosphatidic acid, whose actions are determined by their specific modes of binding. Furthermore, we show that an empty-pocket channel lacking an endogenous phosphoinositide lipid assumes an agonist-like state, even at low temperature, substantiating the concept that phosphoinositide lipids serve as negative TRPV1 modulators whose ejection from the binding pocket is a critical step toward activation by thermal or chemical stimuli.

## Abstract (web summary)

Using cryo-EM, the authors elucidate the mechanisms of TRPV1 regulation by bioactive lipids, namely phosphoinositides and the inflammatory lipid lysophosphatidic acid.

## Main

Foundational studies of transient receptor potential (TRP) channels in the *Drosophila* phototransduction pathway implicated phosphoinositide lipids as key modulatory agents^1,2^. Specifically, both genetic and electrophysiological studies have suggested that hydrolysis of phosphatidylinositol 4,5-bisphosphate (PIP_2_) is a critical step connecting activation of phospholipase C (PLC)-coupled rhodopsin to channel gating. This theme has since been extended to many members of the vertebrate TRP channel family, where both positive and negative modulatory effects of phosphoinositide lipids have been proposed^3^. Although transient receptor potential cation channel subfamily V member 1 (TRPV1) is not strictly a ‘receptor-operated’ channel, its sensitivity to heat and chemical agonists is enhanced by pro-algesic agents, such as bradykinin and nerve growth factor, that activate PLC-coupled metabotropic receptors, consistent with the idea that hydrolysis of phosphoinositides disinhibits the channel to promote gating^4^. Our previous findings support this model by showing that that the vanilloid binding pocket (VBP) is a critical regulatory site that harbors an endogenous phosphoinositide lipid when TRPV1 is in its inactive closed state, and that vanilloid agonists displace this lipid in the course of activating the channel^5,6^.

While physiological studies have shown that multiple phosphoinositide lipid species can inhibit TRPV1 (ref. ^7^), structural analyses have not provided definitive identification of the entity found in the VBP of the inactive channel. Furthermore, the idea that phosphoinositide lipids inhibit TRPV1 is inconsistent with the observation that soluble PIP_2_ analogs with shortened tails enhance, rather than inhibit, TRPV1 activation^8,9^. Also at issue is whether endogenous pro-algesic lipids bind to the same site and mediate their effects by displacing the resident phosphatidylinositol (PI) lipid from the VBP^10^ as has been observed with exogenous lipophilic ligands such as capsaicin and other vanilloids.

In this Article, we address these questions using cryogenic electron microscopy (cryo-EM), which allows us to visualize the occupancy of the VBP and how this relates to the functional state of the channel. We captured several states of TRPV1 ranging from channels in which the VBP is empty to those bearing different bioactive lipids, including distinct phosphoinositide species, brominated phosphoinositide analogs and a pro-algesic lipid, lysophosphatidic acid (LPA). Together, our data show how the VBP accommodates a range of bioactive lipids and how occupancy of the site corresponds to the structural status of the pore.

## Results

### Ejecting the resident PI lipid favors the open state

**Fig. 1:**
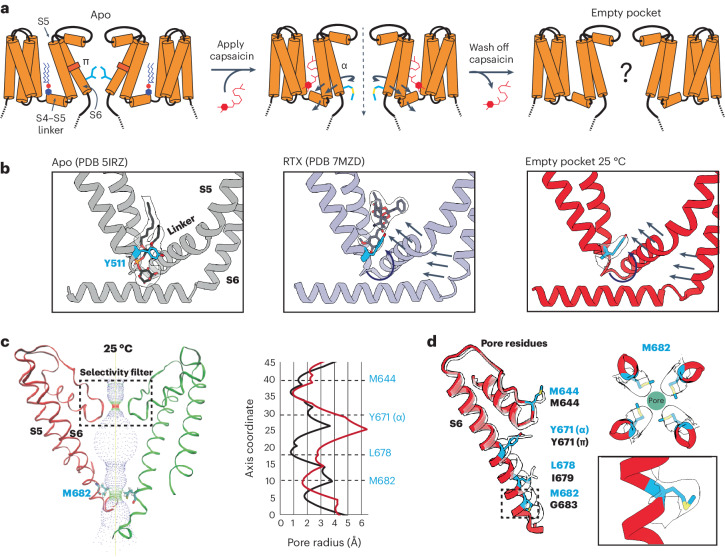
Empty-pocket TRPV1. **a**, Schematic of the capsaicin washout procedure for obtaining empty-pocket TRPV1. **b**, VBP in the apo state (PDB 5IRZ), bound with RTX (PDB 7MZD) or in the empty-pocket state at 25 °C (PDB 8U3L; this study). **c**, Left: pore profile of empty-pocket TRPV1 at 25 °C. Right: pore radius of empty-pocket TRPV1 at 25 °C (red) and apo TRPV1 (black) were determined using the HOLE program. **d**, Key residues lining the channel pore. The red ribbon with blue labels depicts empty-pocket TRPV1 at 25 °C; the transparent ribbon represents apo TRPV1.

To determine the effect of the resident PI lipid on channel gating, we devised a protocol to eject this lipid and render the binding pocket empty (Fig. 1a). First, we used capsaicin to displace the resident lipid from detergent-solubilized channels. We then washed out the competing vanilloid followed by reconstitution of the channel into lipid nanodiscs of defined composition and devoid of phosphoinositide lipids. These samples were applied to grids held at 4 °C or 25 °C (temperatures at which TRPV1 is normally closed) and then taken forward for cryo-EM analysis. The final maps were refined using C4 symmetry and resolved to 2.9 Å and 3.7 Å for the 4 °C and 25 °C samples, respectively. All data collection, processing and validation statistics are shown in Tables 1 and 2.

**Extended Data Fig. 1:**
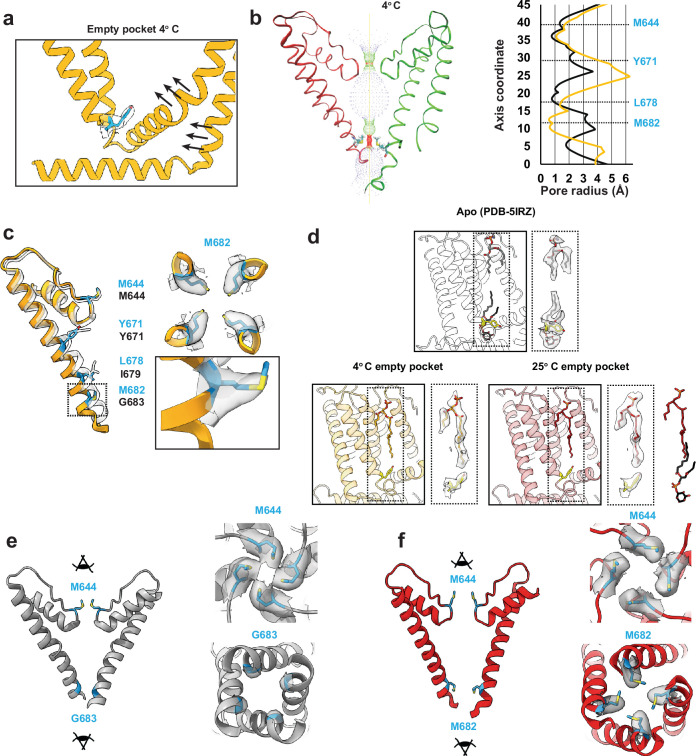
Supplementary TRPV1 empty pocket data. (**a**) Vanilloid pocket with empty-pocket TRPV1 at 4 °C. (**b**) Pore profile for empty-pocket TRPV1 at 4 °C. Pore radius was determined using the HOLE program. Black is apo TRPV1 and orange is empty-pocket TRPV1. (**c**) Key pore residues of empty-pocket TRPV1 (orange) compared to apo TRPV1 (PDB-5IRZ). Density for M682 is shown. (**d**) Comparing the density for an outer leaflet lipid in the apo (PDB-5IRZ) and the empty-pocket states. The resident lipid in PDB-5IRZ (black sticks) and the outer leaflet lipid (dark red sticks) are shown superimposed beside the 25 °C data to highlight the binding overlap of the two lipids. (**e**) Upper methionine restriction (M644) and G683 of apo TRPV1 (PDB-5IRZ). (**f**) Upper methionine restriction (M644) and lower methionine restriction (M682) of TRPV1 in the empty-pocket pocket at 25 °C.

In both cryo-EM structures the vanilloid pocket is, indeed, unoccupied by either a resident PI lipid or capsaicin (Fig. 1 and Extended Data Fig. 1). We thus refer to these structures as ‘empty’ to differentiate from the ‘apo’ structures, in which an endogenous lipid occupies the pocket. Previous functional and structural studies have identified tyrosine 511 (Y511) as a residue whose reorientation toward the vanilloid pocket correlates with ejection of the resident PI lipid upon ligand binding^5,6,11^. Interestingly, we observed that Y511 is flipped toward the binding pocket in these two structures, akin to what is seen in the agonist-bound state, demonstrating that reorientation of this fiducial side chain primarily reflects ejection of the resident lipid (Fig. 1b). The empty space in the vanilloid pocket is now partially occupied by an acyl tail from an annular lipid in the outer leaflet (Extended Data Fig. 1d) that does not reach the bottom of the pocket to restrict Y511 reorientation.

We next asked how these actions affect the ion permeation pathway. Overall, the pathway resembles that of vanilloid-activated channels in which the S4–S5 linker and S6 helices move away from the central axis (Fig. 1c,d and Extended Data Fig. 1a–c). We also see transition of a π-helical turn in S6 to an α-helical configuration (π–α transition), resulting in rotation of the lower half of the S6 helix by one residue. This π–α transition rotates the gating residue L679 away from the central axis with a narrowest restriction now formed by rotation of M682 into the pore. This new restriction resembles that formed by M644 in the selectivity filter (Extended Data Fig. 1e,f). Consistent with a previous study^6^, we see a presumptive Na^+^ ion coordinated to G643 in the selectivity filter, located just below M644, indicating that this configuration, in which methionine residues form the narrowest points in the ion permeation pathway, is conducting. Indeed, consistent with our previous study^6^, the difference in the *B* factor of M682 compared to a nearby residue (A680) is 15.01 Å^2^ for data at 25 °C and 10.57 Å^2^ for data at 4 °C. This is substantially higher than the *B* factor difference in the apo state (~1 Å^2^), which shows that M682 is more dynamic in the α state, and thus suggesting that M682 is sufficiently malleable to pass ions in the α state. Importantly, π–α transitions are seen when agonists, but not antagonists, occupy the VBP^5,6,11^, and these data now show that ejection of the resident lipid is also sufficient to support this transition. Interestingly, the main difference between 4 °C and 25 °C structures was seen in the density of the M682 side chain, which was less well resolved in the latter, implying a more dynamic nature at higher temperature. This contrasts with the π–α transition, which is seen at both temperatures and thus driven primarily by PI lipid ejection and not temperature.

### PI and PIP_2_ favor the closed channel state

**Fig. 2:**
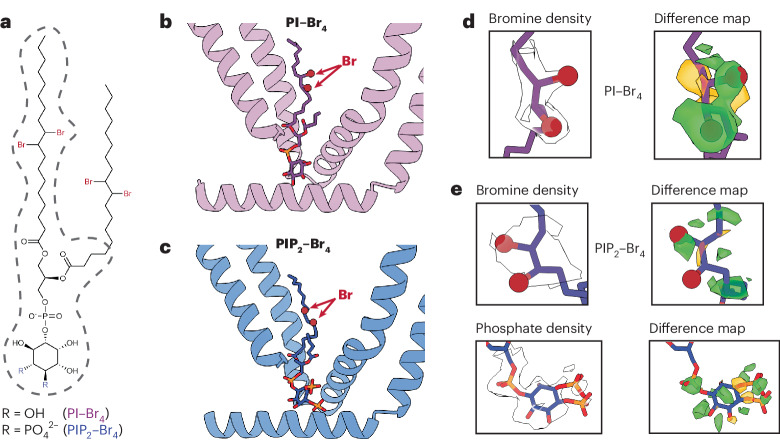
Brominated phosphoinositide binding to TRPV1. **a**, The chemical structure of brominated phosphoinositides used in this study. The part of the lipids with resolvable density is outlined with dashed gray lines. **b**,**c**, VBP bound with PI–Br_4_ (**b**) and PIP_2_–Br_4_ (**c**). **d**,**e**, Density map of key functional groups for PI–Br_4_ (**d**) and PIP_2_–Br_4_ (**e**) with corresponding difference maps. Difference maps were determined by subtracting calculated map of models lacking bromine atoms and the phosphate moieties at 4 and 5 positions of the inositol headgroup from the experimental map (Methods). Both positive (green) and negative (orange) difference densities are shown.

**Extended Data Fig. 2:**
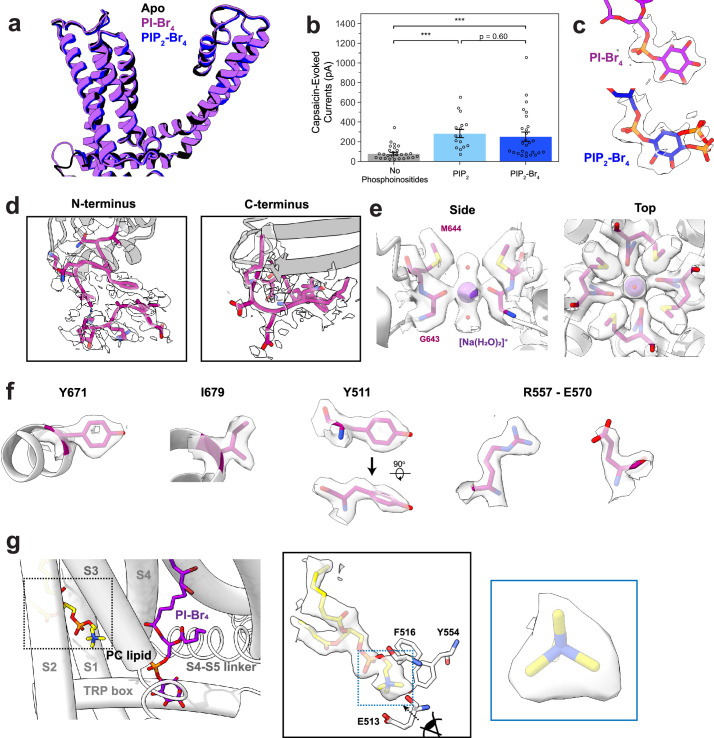
Structural details revealed by the brominated lipid data. (**a**) Superimposition of the transmembrane region of TRPV1 in the apo (black), PI-Br_4_-bound (purple), and PIP_2_-Br_4_-bound (blue) states. (**b**) Patch clamp summary of TRPV1 purified and reconstituted into defined liposomes, in conditions without phosphoinositides (patch clamp recordings, n = 27), with PIP_2_ (n = 16), or PIP_2_-Br_4_ (n = 27) added. Currents were evoked with 10 µM capsaicin at a membrane potential of +120 mV. Hollow circles represent individual data points, bar graph indicates mean ± S.E.M. for the condition. Statistical significance was determined by Kruskal-Wallis test (H = 24.8, p = 4.2 × 10^−6^) and post-hoc Dunn’s test using Bonferroni correction (p = 1.6 × 10^−5^ for No Phosphoinositides vs PIP_2_, p = 4.6 × 10^−4^ for No Phosphoinositides vs PIP_2_-Br_4_, p = 0.60 for PIP_2_ vs PIP_2_-Br_4_), *** p < 0.001. (**c**) Lipid headgroup densities for PI-Br_4_ and PIP_2_-Br_4_. One monomer is shown for clarity. (**d**) Higher resolution for the N-terminus and C-terminus of the PI-Br_4_ consensus map allows for further modeling of the N-terminus (starting at residue 177) and the remaining C-terminus of the truncated TRPV1 construct (up to residue 764). (**e**) Captured density at the selectivity filter in the PI-Br_4_ consensus map models well to a [Na(H_2_O)_2_]^+^ cation coordinated to G643. (**f**) Well-defined densities for key residues as revealed in the PIBr_4_ consensus map. (**g**) Lipid density in a binding pocket within the S1 to S3 transmembrane helices shows well-defined features for a PC headgroup, importantly the tri-lobular of the quaternary amine group. Data shown is from the PI-Br_4_ data in Conformation 1. Source data

**Extended Data Fig. 3:**
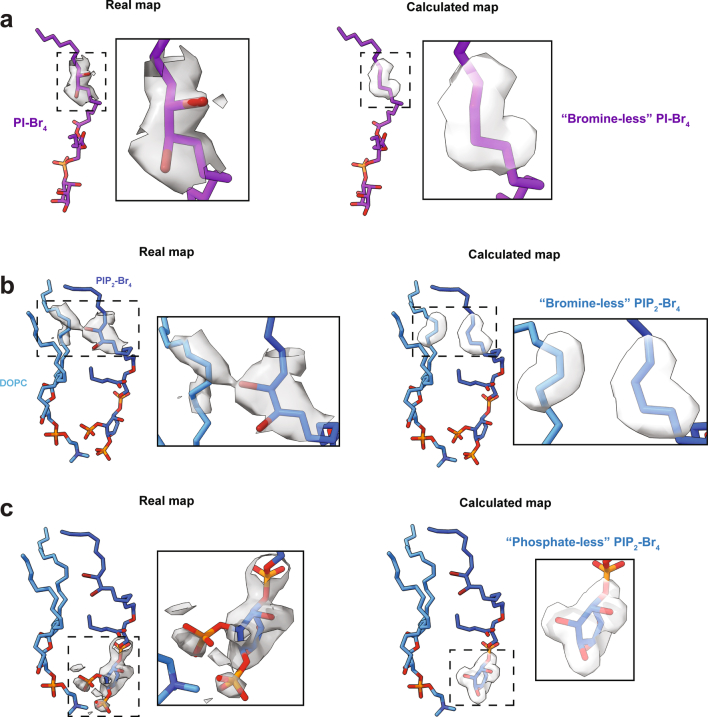
Real and calculated maps used for difference map analysis. (**a**) Maps used for PI-Br_4_ analysis. (**b**) Maps used for the bromine analysis for PIP_2_-Br_4_. (**c**) Maps used for the headgroup analysis for PIP_2_-Br_4_. Insets show close ups of the maps.

In all cryo-EM structures of TRPV1 reported so far, the resident lipid shows clear features of a phosphoinositide, but the unresolved number of phosphate moieties on the inositol ring makes assignment to a specific species ambiguous. Moreover, the VBP is sufficiently flexible to accommodate diverse phosphoinositide species. Indeed, we have previously shown that PI or PIP_2_ inhibits TRPV1 when reconstituted into proteoliposomes^7^, suggesting that both species can bind to the VBP and stabilize a closed state. To resolve this question, we exploited our ability to generate TRPV1 protein with an empty vanilloid pocket, into which we introduced tetrabrominated analogs of PI or PIP_2_ (PI–Br_4_ and PIP_2_–Br_4_, respectively) as contrast-enhancing probes to distinguish these lipids from the other, unbrominated lipids in the nanodiscs (Fig. 2a). Previous work has established that brominated lipids are faithful analogs of the unsaturated lipids from which they are synthesized^12^. Indeed, PIP_2_ or PIP_2_–Br_4_ support TRPV1 function equivalently in reconstituted proteoliposomes (Extended Data Fig. 2b). In these high-resolution structures (2.2–2.4 Å resolution) (Extended Data Fig. 2), we were able to identify the bromine atoms within the oleoyl tails of the lipids by visually comparing the resulting reconstructions against those previously determined for samples containing endogenous lipids (Fig. 2 and Extended Data Fig. 3). The well-resolved inositol headgroup of the bound lipids showed the expected differential location of phosphate moieties, most notably at the 4 and 5 positions for PIP_2_. Furthermore, conformations of both PI- and PIP_2_-reconstituted channels resemble the apo channel containing a native resident lipid. Together, these data demonstrate that the vanilloid site can accommodate PI or PIP_2_, where they act as negative regulatory factors to stabilize the closed state.

### Soluble short-chain PIP_2_ is a partial TRPV1 potentiator

**Fig. 3:**
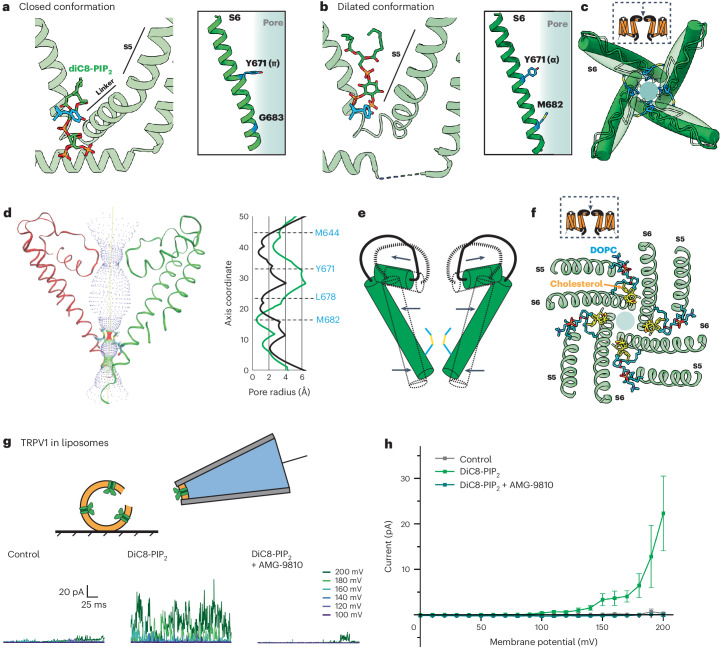
DiC8-PIP2 is a partial potentiator of TRPV1 activity. **a**,**b**, VBP with diC8-PIP_2_ bound in the closed conformation (**a**) and the dilated conformation (**b**). **c**, Top-down view of the TRPV1 pore in the closed conformation (transparent green) and the dilated conformation (dark green). M644 (blue) of the selectivity filter is highlighted. **d**, Left: pore profile of TRPV1 in the dilated conformation. Right: pore radii of closed (black) and dilated (green) states. **e**, Schematic of the pore movements demonstrating the dilation of the upper portion of the pore and constriction of the lower portion. **f**, Top-down view of TRPV1 showing the binding of DOPC (blue) and cholesterol (yellow). **g**, Top: schematic showing excised inside-out patch clamp recording configuration. Bottom: sample TRPV1 currents evoked by application of control (bathing) solution, 100 µM DiC8-PIP2, or 100 µM DiC8-PIP2 plus 10 µM antagonist AMG-9810. **h**, Summary of current–voltage relationships showing that DiC8-PIP2 reliably activates TRPV1. Data are graphed as mean ± s.e.m., patch clamp recordings *n* = 10. Source data

Physiological effects of phosphoinositides are often examined using soluble analogs of PIP_2_ with shortened acyl tails (such as diC8-PIP_2_) to facilitate cellular application during electrophysiological or imaging experiments. In the case of TRPV1, perfusion of diC8-PIP_2_ onto TRPV1-expressing cells has been shown to enhance channel activity, suggesting that phosphoinositide lipids serve as positive channel regulators^8,13^, conflicting with the idea that activation of PLC-coupled receptors potentiates TRPV1 activity through hydrolysis of PIP_2_ to release the channel from phosphoinositide inhibition^4^. Considering this controversy, we were interested in determining whether diC8-PIP_2_ and full-length PIP_2_ interact with TRPV1 in a similar manner. We therefore obtained cryo-EM data for samples in which diC8-PIP_2_ was provided to the empty-pocket TRPV1 preparation. Using focused classification, we were able to identify two key states, one corresponding to a closed state (3.0 Å) and the other having a dilated selectivity filter (3.6 Å) (Fig. 3 and Supplementary Video 1).

**Extended Data Fig. 4:**
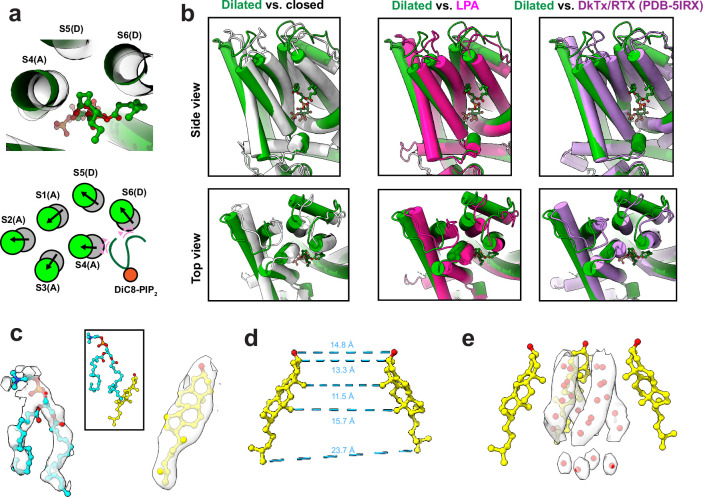
TRPV1 bound with diC8-PIP_2_ in the dilated state. (**a**) Top: Top-down view of diC8-PIP_2_ (green sticks and balls) and surrounding helices. Grey ghost tubes are TRPV1 bound with diC8-PIP_2_ in the closed state and green tubes are in the dilated state. Bottom: schematic comparing the helical positions between the closed and dilated states. Pink triangles indicate the steric overlap between the diC8-PIP_2_ molecule in the dilated state and the transmembrane helices of TRPV1 in the closed state. (**b**) Comparisons of the dilated state of TRPV1 with diC8-PIP_2_ bound and other states. (**c**) Densities for cholesterol and a lipid (modeled as DOPC) that is found in a cleft between the S6 and S5 helices of adjacent monomers (see Fig. 3). (**d**) Interatom distances between two opposite cholesterol molecules demonstrating the pore radius formed by cholesterol. (**e**) Extra densities within the pore that are modeled as ordered water molecules due to the hydrophobic effect. 3 of the 4 cholesterol molecules are shown for clarity.

In the closed state, channels with diC8-PIP_2_ adopt a conformation like that containing full-length PIP_2_ (Fig. 3a), with the acyl chains of the bound lipid preventing reorientation of Y511 or inward movement of the S4–S5 linker. In the dilated state, diC8-PIP_2_ sits higher up in the vanilloid pocket such that the inositol headgroup sits above Y511, which is reorientated toward the pocket (Fig. 3b). Moreover, Y671 adopts an α-helical conformation and M682 and is oriented toward the pore axis. These features are hallmarks of a channel in which the resident PI lipid has been displaced (that is, the pocket is empty or occupied by a vanilloid agonist). However, there are some differences between the diC8-PIP_2_ dilated state and a typical vanilloid agonist-bound structure. Most notably, in the diC8-PIP_2_ dilated state the angle of the S6 helix is more dramatically tilted such that the voltage sensor-like domain (S1–S4, VSLD) is pivoted outward away from the central axis, and the upper region of the ion permeation pathway, including the selectivity filter, is expanded and stabilized by cholesterol and another membrane lipid (probably 1,2-dioleoyl-*sn*-glycero-3-phosphocholine (DOPC)) (Fig. 3e,f and Extended Data Fig. 4). At the same time, pivoting of the S6 helix makes the new restriction site formed by M682 narrower. We and others have previously suggested that outward tilting of the VSLD is coupled to movements of the S6 helix that facilitate gating^6,14^. Thus, this dilated structure is consistent with the functional effects of diC8-PIP_2_, which enhances capsaicin sensitivity, but by itself activates the channel only at highly depolarized states (for example, +150 mV membrane potential)^8,9^ (Fig. 3g–i).

### Channel activation by LPA

LPA is an endogenous bioactive lipid that mediates a host of physiologic responses, including cellular migration and proliferation, inflammation and pain^15^. While LPA is known to activate metabotropic receptors (G-protein-coupled receptors), it has also been shown to activate or potentiate ionotropic receptors, including TRPV1 (refs. ^10,15,16^). Indeed, LPA activates recombinant TRPV1 channels in reconstituted liposomes^7^ and elicits TRPV1-dependent pain-related behavior in mice^10^. Thus, we are interested in determining how this endogenous pro-algesic lipid binds to TRPV1, and whether and how this involves interaction with the vanilloid site.

**Fig. 4:**
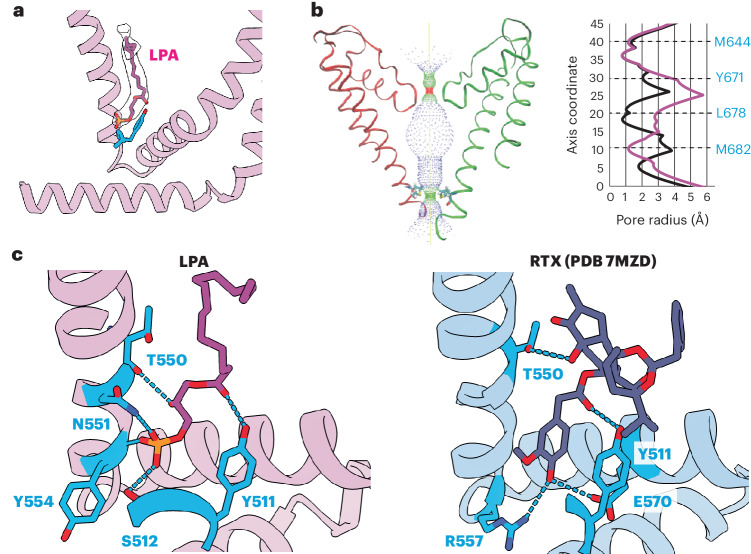
LPA binding to TRPV1. **a**, VBP with LPA bound. **b**, Left: pore profile of TRPV1 with LPA bound. Right: pore radii show profiles for LPA (magenta) compared to the corresponding apo state (black). **c**, Molecular interactions between ligand headgroups and TRPV1. Residues that form electrostatic interactions with ligand functional groups (within 3.5 Å) for LPA and RTX (PDB 7MZD) are shown in blue.

**Fig. 5:**
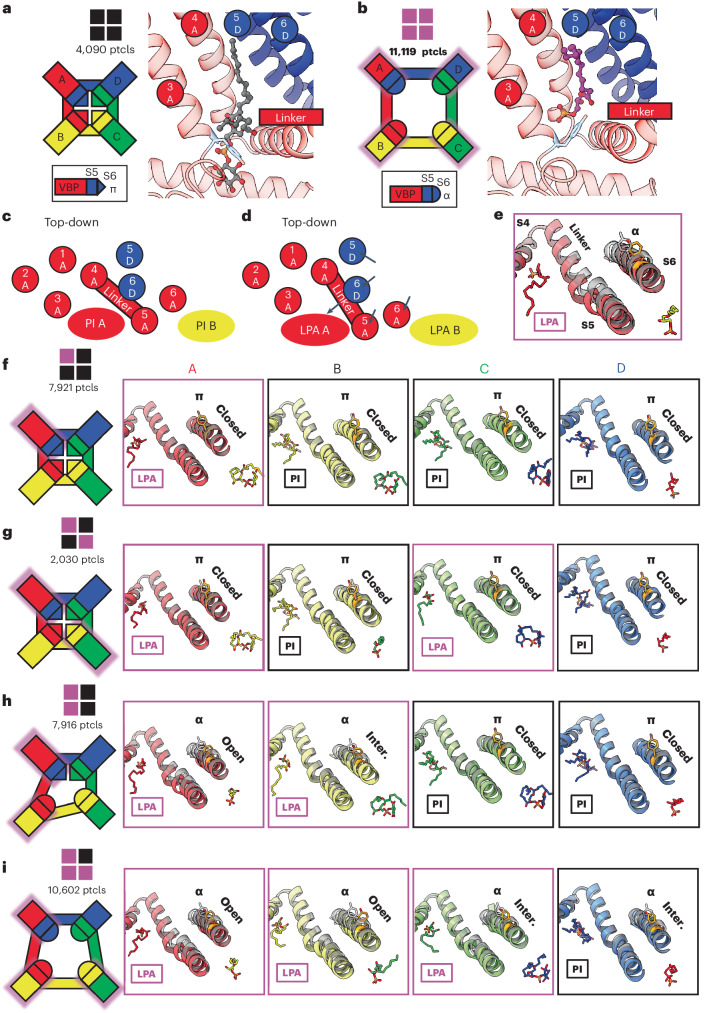
Substoichiometric states of LPA binding. **a**, The closed configuration of TRPV1 with all four subunits occupied by PI lipid. Particle numbers (ptcls) are shown. **b**, The open configuration of TRPV1 with LPA bound to all four subunits. The VBPs of the A monomers are shown to demonstrate the domain-swap architecture that comprises the VBP (S3, S4 and S4–S5 from A; S5 and S6 from D). **c**,**d**, Schematic of the binding pocket from a top-down view highlighting the domain-swap architecture and helical movements between the closed (**c**) and open (**d**) conformations. **e**, Structural changes associated with the binding of LPA compared to apo (transparent black). **f**–**i**, Substochiometric states of LPA binding with monomers bound with 1 LPA (**f**), 2 LPA in opposite pockets (**g**), 2 LPA in neighboring pockets (**h**) and 3 LPA (**i**). The leftmost panels show a cartoon representation of the TRPV1 tetramer indicating the functional state of each VBP monomer. Monomers are labeled anticlockwise; LPA-occupied monomers are shadowed with magenta. The functional state of the pore is indicated as ‘closed’ (π helix at Y671), ‘open’ (α helix at Y671 with S6 moved away from the pore to the same extent as fully occupied LPA) and ‘intermediate’ (‘inter.’, α helix at Y671 with S6 positioned between that of apo and fully occupied LPA).

**Extended Data Fig. 5:**
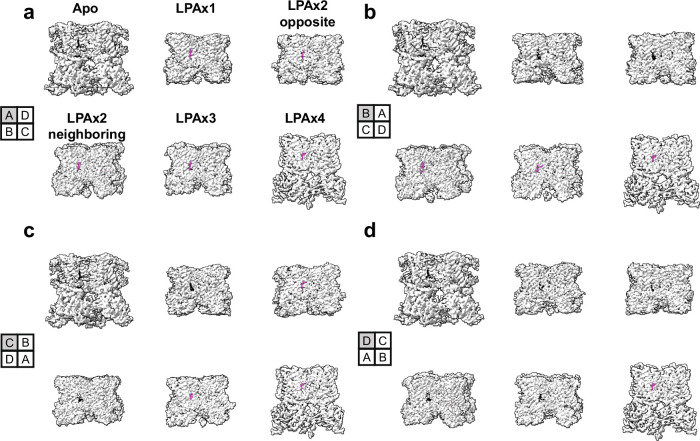
Density maps of TRPV1 bound with 0–4 LPA. Each subpanel. (**a-d**) shows TRPV1 rotated 90 °Counterclockwise starting with pocket A (**a**) and ending with pocket D (**d**). Density corresponding to the resident phosphoinositide lipid is shown as black and density corresponding to LPA is shown as magenta.

To visualize the lipid–channel complex, we added LPA to nanodisc-reconstituted TRPV1 and determined a consensus cryo-EM map to a resolution of 3.0 Å. Through symmetry expansion and focused classification, we captured LPA bound within the vanilloid pocket in multiple stoichiometries, ranging from apo to all four subunits occupied (Figs. 4 and 5, and Extended Data Fig. 5). LPA displaces the endogenous PI lipid by occupying the upper region of the pocket that binds aliphatic tails (Fig. 4a). As in the case of vanilloid agonists, Y511 flips toward the VBP so that its hydroxyl group forms an H-bond with the ester carbonyl of the LPA tail (Fig. 4c). Moreover, S512, T550 and Y554 engage in H-bond interactions with the phosphate moiety of LPA, while the free hydroxyl of the glycerol backbone interacts with N551. Further comparison between bound LPA and the ultrapotent vanilloid agonist resiniferatoxin (RTX) shows that both form H-bonds with Y511 and T550 (Fig. 4c). An important difference is that the 4-OH of the RTX vanilloid moiety engages in a bridging H-bond network between R557 and E570, two residues that form a salt bridge when TRPV1 is fully opened^5^. This reflects the fact that the RTX 4-OH sits lower into the pocket, closer to R557 and E570, compared to the LPA headgroup. Therefore, the RTX headgroup may be better suited for priming this salt bridge interaction as compared to LPA, which may help to explain the greater efficacy of RTX as an agonist. Together, these structures demonstrate that LPA utilizes similar molecular interactions as vanilloid agonists, such as RTX or capsaicin, for binding to TRPV1 and initiating gating. In the LPA-bound structure, the selectivity filter remains unchanged from the resting state, but we do see that S6 adopts an α helical configuration, resulting in rotation of the lower half of the S6 helix by one residue (Fig. 4b), consistent with LPA acting as an agonist.

### LPA substates reveal allosteric gating

In addition to the two structures described above, we also captured structures of TRPV1 with substoichiometric LPA binding, including one LPA, two LPAs bound to either neighboring or opposite subunits, or three LPAs (Fig. 5 and Extended Data Fig. 5). With resting and fully liganded structures representing closed and open configurations of the pore (S6) helices, respectively (Fig. 5a,b), we examined intermediate conformational states associated with substochiometric LPA binding (the four subunits are denoted as A through D in a counterclockwise orientation as viewed from the extracellular face).

**Extended Data Fig. 6:**
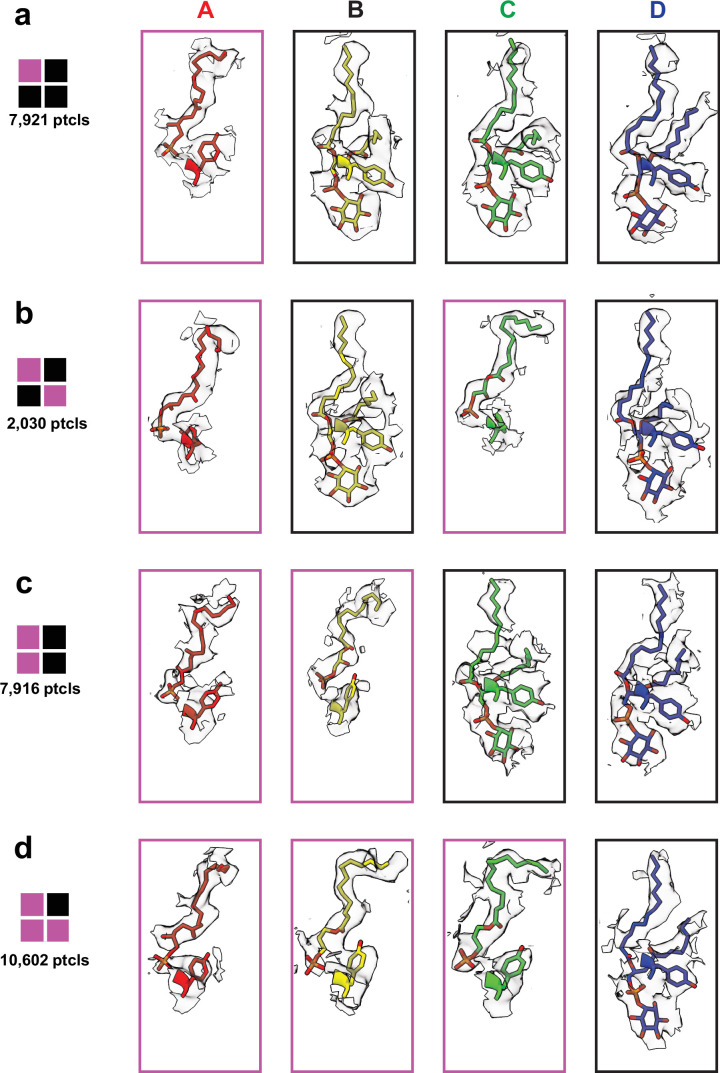
LPA sub-stoichiometric ligand densities. Densities for Y511 and LPA or the resident lipid are shown for each monomer. LPA = magenta; resident lipid = black. (**a**) LPAx1. (**b**) LPAx2 opposite. (**c**) LPAx2 neighboring. (**d**) LPAx3.

**Extended Data Fig. 7:**
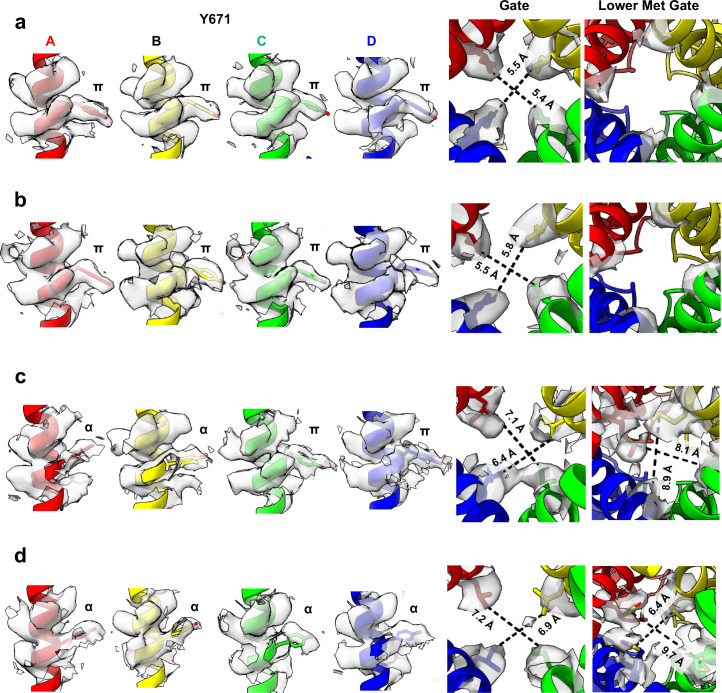
Densities of key residues lining the pore of the sub-saturating states. (**a**) LPAx1. (**b**) LPAx2 in opposite pockets. (**c**) LPAx2 in neighboring pockets. (**d**) LPAx3. Interatom distances of the closest non-hydrogen atoms for the gating residues and the lower methionine filter are shown.

Binding of one LPA to subunit A does not induce an appreciable conformational change to TRPV1 other than replacement of the resident PI lipid and flipping of Y511, with no change to the pore configuration (Fig. 5f and Extended Data Fig. 6a). The same is true when a second LPA binds to the opposite subunit (A and C) (Fig. 5g and Extended Data Fig. 6b). However, when the second LPA binds to an adjacent subunit (A and B), we see that gating transitions occur in the S6 helices owing to ejection of the resident PI lipid from both neighboring pockets, which communicate by virtue of domain-swap architecture (Fig. 5h and Extended Data Fig. 6c). Specifically, we see that one S6 helix (S6-A) is in the open configuration and the neighboring helix (S6-B) is in an intermediate position between open and closed because the resident PI lipid in the C subunit prevents full backward movement of S6-B (Extended Data Fig. 7). S6-C and S6-D remain in closed configurations (Fig. 5h). The binding of the third LPA induces all four S6 helices to adopt the π–α transition, with the S6-B and S6-C in the open configuration and the S6-D and S6-A helices in intermediate positions (Fig. 5i and Extended Data Fig. 6d). Together, these observations are consistent with a model in which ligand binding induces allosteric gating movements in the adjacent VBP through domain-swap interactions.

How does binding of LPA promote movement of S6? Reorientation of Y511 toward the VBP is a key step, allowing L574 on the S4–S5 linker to partially occupy the now vacant space, thereby facilitating movement of the S4–S5 linker toward the VBP (Extended Data Fig. 6c and Supplementary Videos 2 and 3). Movement of the S4–S5 linker enables the S5 and S6 helices to move away from the pore axis while providing space to accommodate a π–α helix transition in S6, which is associated with the loss of hydrophobic interactions between M682 and the S4–S5 linker (Extended Data Fig. 6b). At the same time, reorientation of Y511 is necessary, but not sufficient, to facilitate gating movements, consistent with the observation that TRPV1 antagonists also induce similar reorientation of Y511 (refs. ^5,11^).

While the fully liganded structures obtained with LPA or RTX are similar, the intermediate configurations associated with substoichiometric ligand binding are noticeably different. For example, movements of the S4–S5 linker are smaller when RTX is the agonist, and the S6 helix does not move until all four subunits are occupied^6^. One caveat to comparing LPA and RTX substates is that those observed with RTX were obtained under low-sodium conditions. Indeed, LPA-bound substates may be more physiologically relevant because they were obtained under normal ionic conditions, and LPA is an endogenous, physiologically relevant ligand.

From this analysis, we infer that the first LPA binds to TRPV1 randomly, but such binding increases the affinity for binding to the adjacent versus the opposite monomer. This behavior is supported by the observation that the particle numbers of two LPA molecules bound to opposite versus neighboring subunits are about 1:4, consistent with increased propensity for binding to an adjacent monomer.

## Discussion

Bioactive lipids are believed to play important roles as pro-algesic agents that contribute to inflammatory pain, in part by enhancing TRPV1 function. However, their site(s) of action and relationship to gating has not been directly visualized. Our structures now show that LPA, an important inflammatory lipid, binds to the VBP and displaces the resident PI lipid, further validating this site as a key regulatory locus for the action of both endogenous and exogenous lipophilic agents. While the VBP can accommodate structurally diverse ligands, including agonists and antagonists, their precise location dictates the functional outcome, as exemplified by our analysis of diC8-PIP_2_. In this case, one ligand can bind to the same pocket but with its inositol headgroup located in two distinct positions associated with closed or dilated pore states. This detailed structural insight resolves a prior conundrum by explaining how diC8-PIP_2_ and full-length PIP_2_ can mediate different effects on TRPV1 function. This, together with our observation that the empty-pocket channel is in an agonist-like state, substantiates the model in which the resident PI lipid stabilizes the closed state, and its removal favors the open state. Importantly, we also find that the empty-pocket channel is open at temperatures well below its normal thermal activation threshold, supporting the idea that ejection of the resident lipid is also a critical step in heat-evoked TRPV1 activation^5,17^. This is also consistent with a more recent observation that the heat-activated TRPV3 channel lacks a resident lipid in a region analogous to that of the VBP in TRPV1 (ref. ^18^). Interestingly, some ligands, such as the competitive vanilloid antagonist capsazepine, eject the resident lipid but counteract this effect by stabilizing the closed state. This suggests that, in addition to lipid ejection, the gating state depends on specific ligand–channel interactions.

**Extended Data Fig. 8:**
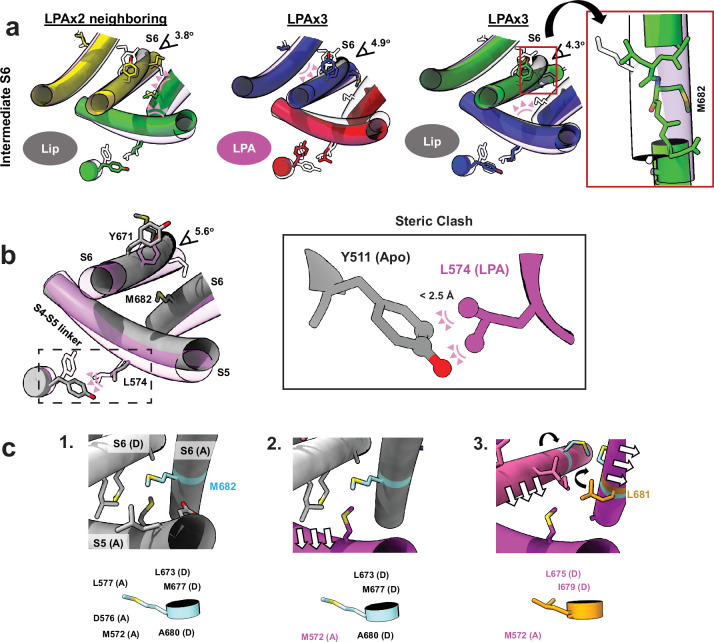
Intermediate states and mechanism of allostery. (**a**) Intermediate states of the S6 helices. Steric clashes preventing full opening of the S6 helix are shown as pink triangles. Angles of opening are shown as a measure of the intermediate state. For LPAx3, the S6-C helix is distorted from a typical α-helical structure. (**b**) Steric clash between the superimposed position of Y511 in the closed state (grey) and L574 in the LPAx4 state (magenta) shows that the inward movement of Y511 is necessary for open transitions to occur. Boxed figure shows a zoom-in of the steric clash represented as pink pseudobonds (<2.5 Å distance). (**c**) Proposed mechanism for the LPA-induced opening of TRPV1 as the channel goes from apo state (grey) to one completely occupied by LPA (magenta). Residues in contact with M682 (< 5.0 Å) as it switches positions with L681 due to the π-α helix transition are highlighted. 1. M682 maintains contact with several hydrophobic residues in the apo state. 2. The S4-S5 linker moves inward toward the pocket because of Y511 flipping toward the pocket with LPA bound; the space around M682 opens. 3. S5 and S6 dilate away from the pore axis because of the void created by the moving of the S4-S5 linker; M682 is able to rotate towards the pore axis and the π-α transition occurs.

Previous electrophysiological studies showed that the charge-reversal mutation, K710D, abrogates LPA-evoked responses, leading to the conclusion that K710, a residue located on the periphery of TRPV1 at the membrane–protein interface, is key in mediating the binding of LPA^10^. However, our cryo-EM data place the binding of LPA in the VBP, about 25 Å away from K710. We propose that K710 is part of a basic tunnel through which LPA accesses the VBP. Indeed, when the TRPV1 structure is subjected to CAVER analysis, a tunnel is seen at the surface of the inner membrane that connects K710 to the VBP (Extended Data Fig. 8) and which is composed of several basic residues. The notion that this tunnel represents a conduit for LPA is consistent with the observation that channel activation is most robust when LPA is applied to excised inside-out membrane patches, providing direct access to the intracellular face of the membrane^10^. Thus, K710 may serve as a guiding residue directing LPA into this access tunnel by interacting with the negative charge of the phosphate headgroup on LPA.

More generally, we and others have shown that lipid regulation is a common feature of many TRP channel family members^3^. While the interaction of phosphoinositide lipids with the TRPV1 VBP is perhaps the best described, structural and functional studies suggest that other binding sites may exist. One such example is a proposed interaction between PIP_2_ and positively charged residues in the cytoplasmic carboxy-terminal tail^19^; another is a putative site between neighboring subunits of TRPM8, where PIP_2_ binding may enhance channel activity^20^. The structural details and consequences of phosphoinositide binding to these or other sites remain unresolved.

## Methods

### Materials

Reagents were purchased from Sigma-Aldrich unless noted below. The 18:1 LPA ((2-hydroxy-3-phosphonooxypropyl) (*Z*)-octadeca-9-enoate) was purchased from Cayman Chemical Company for cryo-EM sample prep and diluted in dimethyl sulfoxide (DMSO). DiC8-PIP_2_ (1,2-dioctanoyl-*sn*-glycero-3-phospho-(1′-myo-inositol-4′,5′-bisphosphate)) was purchased from Avanti Polar Lipids and diluted in aqueous buffer before cryo-EM or electrophysiology. Soy polar lipid extract, di18:1 PI, and di18:1 PI(4,5)P_2_, DOPC, 1-palmitoyl-2-oleoyl-*sn*-glycero-3-phospho-(1′-rac-glycerol) (POPG), 1-palmitoyl-2-oleoyl-*sn*-glycero-3-phosphoethanolamine (POPE), 1-palmitoyl-2-oleoyl-glycero-3-phosphocholine (POPC) and sphingomyelin were also purchased from Avanti Polar Lipids. Bio-beads SM2 was purchased from Bio-Rad. Freestyle 293 Expression Medium and Expi 293 Expression Medium were purchased from Gibco, along with Expi-293F cells and the ExpiFectamine-293 Transfection Kit. Sf9 and insect cell culture media were purchased from Expression Systems. Fetal bovine serum was purchased from PEAK, and bovine calf serum was purchased from HyClone. HEK293 GnTI^−^ cells and HEK293T cells were purchased from ATCC. DH5α competent cells were purchased from New England Biolabs. Quantifoil R1.2/1.3 Au 300 mesh grids were purchased from Quantifoil Micro Tools GmbH.

### Brominated lipid synthesis

The 3-((9,10-dibromooctadecanoyl)oxy)-2-(((9,10-dibromo-octadecanoyl)oxy)methyl)propyl ((1*S*,2*R*,3*R*,4*S*,5*S*,6*R*)-2,3,4,5,6-pentahydroxycyclohexyl) phosphate (PI–Br_4_) and (1*R*,2*R*,3*S*,4*R*,5*R*,6*S*)-4-(((3-((9,10-dibromooctadecanoyl)oxy)-2-(((9,10-dibromo-octadecanoyl)oxy)methyl)propoxy)oxidophosphoryl)oxy)-3,5,6-trihydroxycyclohexane-1,2-diyl bis(phosphate) (PIP_2_–Br_4_) were synthesized from PI and PIP_2_, respectively using Br_2_ as previously described^12^. Then, 0.5 mg di18:1-PI(4,5)P_2_ or di18:1-PI was dissolved in 0.5 ml CHCl_3_ and stirred on ice in a glass vial. Br_2_ (stoichiometric with the number of double bonds in the lipid) was added to the vial with a glass syringe. The vial was flushed with argon and sealed. The reaction was allowed to continue in the dark with stirring for 1 h. Solvent and any excess bromine was removed by application of vacuum in the dark overnight. Brominated lipids were stored at −80 °C until use.

### Protein purification and nanodisc reconstitution

Recombinant minimal functional rat TRPV1 (residues 110-603 and 627-764) with a maltose-binding protein (MBP) tag was expressed in HEK293 GnTI^−^ cells and purified as previously described^21^ with the following general modifications. HEK293 GnTi^−^ cells were transfected with a baculovirus system for 48 h before collecting and freezing the resulting cell pellet. The cell pellet was suspended in Buffer A containing 25 mM HEPES (pH 7.5), 150 mM NaCl and 0.4 mM tris(2-carboxyethyl)phosphine (TCEP)·HCl. The suspension was diluted twofold with Buffer B containing 80 mM HEPES (pH 7.5), 150 mM NaCl, 0.5 mM TCEP·HCl, 20% glycerol and 29.4 mM dodecyl maltoside (DDM) and allowed to incubate at 4 °C for 2.5 h. The homogenate was centrifuged at 100,000*g* in a Beckman LE-80 Ultracentrifuge for 45 min. The supernatant was applied to an amylose column and washed with 5× column volumes of Column Buffer (Buffer B diluted 50-fold with Buffer B), and then TRPV1 was eluted with Column Buffer containing 20 mM maltose. Protein was then concentrated for nanodisc assembly.

The membrane scaffold protein, MSP2N2, for nanodisc reconstitution was expressed in *Escherichia coli* as previously described^5^. Nanodisc reconstitution of purified minimal TRPV1 with soybean polar lipids or a defined lipid composition (purchased from Avanti Polar Lipids) was performed following the protocol described previously^5^ with the following modifications. Nanodiscs were assembled with a 1:20:674 ratio of TRPV1–MBP:MSP2N2:lipid. Lipids were combined with MSP2N2 for 30 min on ice before adding TRPV1–MBP to this mixture for another 30 min. Biobeads (0.5 g ml^−1^) were added to the mixture and allowed to incubate overnight at 4 °C with gentle rotation. Tobacco etch virus (TEV) protease (27:1 w:w TRPV1–MBP:TEV) was added to the mixture for 3 h before purifying the nanodiscs using size-exclusion chromatography (AKTA) using a Superdex 200 column. The TRPV1-nanodisc peak was concentrated to 2.1 mg ml^−1^ for cryo-freezing on grids.

Specific deviations from the general scheme above for individual samples are given in subsequent sections.

#### Empty pocket

Five micromolar capsaicin in column buffer was applied to the TRPV1–MBP-bound amylose column for 30 min. The column was washed ten times with 3× column volume. Protein was eluted and then reconstituted into defined-lipid nanodiscs containing (by mol%) 8% cholesterol, 36.8% POPG and 55.2% DOPC. Samples were immediately taken for cryo-EM grid preparation after size-exclusion chromatography.

#### Brominated phosphoinositides

Samples were prepared like the empty-pocket prep. Nanodiscs were prepared using a defined-lipid composition containing 8% cholesterol, 10% brominated phosphoinositide (PI–Br_4_ or PIP_2_–Br_4_), 32.8% POPG and 49.2% DOPC.

#### DiC8-PIP_2_

Sample was prepared using the empty-pocket prep. DiC8-PIP_2_ was applied to sample and then to grid as stated below.

#### LPA

Sample was prepared using the standard protocol with nanodiscs containing soybean polar lipids (that is, resident lipid was not removed).

### Cryo-EM sample preparation and data acquisition

To prepare cryo-EM grids, 3 μl TRPV1-nanodiscs was applied to glow-discharged Quantifoil R1.2/1.3 Au 300 mesh grids covered in holey carbon film (Quantifoil Micro Tools GmbH) and blotted with Whatman 1 filter paper on a Vitrobot Mark IV (FEI Company) with a 4.5 s blotting time, 4 blot force and 100% humidity, and subsequentially plunge-frozen in liquid ethane cooled by liquid nitrogen. Sample was imaged with a Titan Krios microscope (ThermoFisher FEI) operated at 300 kV and equipped with a post-column Bio Quantum energy filter with zero-loss energy selection slit set to 20 eV and a K3 camera (Gatan) either at the University of California, San Francisco (UCSF) or using the Cryo-EM Consortium at Stanford SLAC (S^2^C^2^) as stated below. Data collection was carried out with SerialEM software^22^. The detailed collecting parameters, including dose rate, total dose, total frames per movie stack and so on, are summarized in Tables 1 and 2. Specific conditions not stated above for each of the samples are described below.

**Table 1:** Cryo-EM data collection, refinement and validation statistics

	Empty-pocket 4C (EMDB-41864)(PDB 8U3J)	Empty-pocket 25C (EMDB-41866) (PDB 8U3L)	PI–Br_4_ consensus (EMDB-41879) (PDB 8U4D)	PI–Br_4_ conformation 1 (EMDB-41855) (PDB 8U3A)	PI–Br_4_ conformation 2 (EMDB-41857) (PDB 8U3C)	PIP_2_–Br_4_ (EMDB-41873) (PDB 8U43)	DiC8-PIP_2_ dilated (EMDB-41848) (PDB 8U30)	DiC8-PIP_2_ closed (EMDB-41847) (PDB 8U2Z)
**Data collection and processing**								
Magnification	130,000×	130,000×	130,000×	130,000×	130,000×	105,000×	105,000×	105,000×
Voltage (kV)	300	300	300	300	300	300	300	300
Electron exposure (e^−^ Å^−^^2^)	60	60	47.2	47.2	47.2	45.8	45.8	45.8
Defocus range (μm)	0.5–2.0	0.5–2.0	0.5–2.0	0.5–2.0	0.5–2.0	0.5–2.0	0.5–2.0	0.5–2.0
Pixel size (Å)	0.68	0.68	0.644	0.644	0.644	0.835	0.835	0.835
Symmetry imposed	C4	C4	C4	C1	C1	C4	C4	C4
Initial particle images (no.)	5,90,647	8,01,469	8,08,402	8,08,402	8,08,402	10,89,955	37,96,009	37,96,009
Final particle images (no.)	37,836	39,183	2,63,637	7,15,030	3,39,518	10,89,955	2,17,900	26,265
Map resolution (Å)	2.9	3.7	2.2	2.3	2.3	2.4	3	3.6
FSC threshold	0.143	0.143	0.143	0.143	0.143	0.143	0.143	0.143
Map resolution range (Å)	2.9–6.0	3.5–6.0	1.7–6.6	2.3–3.0	2.3–3.2	1.5–6.0	3.2–5.5	2.8–30
**Refinement**								
Initial model used (PDB code)	7MZD	7MZD	7L2P	7L2P	7L2P	7L2P	7MZD	7L2P
Map sharpening *B* factor (Å^2^)	NA	NA	NA	−70.5	−69.9	−114.7	−112.4	−113.5
Model composition								
Nonhydrogen atoms	17,493	17,493	18,311	3,431	3,459	18,591	17,341	17,045
Protein residues	2,136	2,136	2,236	405	405	2,224	2,112	2,040
Ligands	5	5	5	3	4	9	5	13
*B* factors (min/max/mean Å^2^)	34.88/160.73/92.94	18.11/125.8/59.01	17.01/91.55/48.71	34.50/128.78/77.96	25.26/49.84/33.92	38.70/119.38/76.44	24.23/125.10/70.79	0.12/109.42/34.69
Protein	58.52/67.79/58.76	29.17/48.73/30.09	30.53/31.52/30.79	20.00/58.84/38.32	20.08/38.76/36.56	20.00/63.27/42.00	25.45/52.33/32.72	9.54/39.93/34.72
Ligand								
Root mean square deviations								
Bond lengths (Å)	0.28	0.28	0.29	0.38	0.39	0.32	0.28	0.3
Bond angles (°)	0.5	0.47	0.49	0.55	0.56	0.55	0.48	0.55
**Validation**								
MolProbity score	2	2	2	1.5	1	1.5	2	1
Clashscore	5	5	2	1	1	1	6	8
Poor rotamers (%)	0	0	5	0	0	4	0	1
Ramachandran plot								
Favored (%)	95	94	94	94	95	94	94	92
Allowed (%)	5	6	6	6	5	6	6	8
Disallowed (%)	0	0	0	0	0	0	0	0

NA, not applicable.

**Table 2:** Cryo-EM data collection, refinement and validation statistics

	LPAx0 (EMDB-40941) (PDB 8T0E)	LPAx1 (EMDB-40949) (PDB 8T0Y)	LPAx2 opposite (EMDB-40951) (PDB 8T10)	LPAx2 neighboring (EMDB-41005) (PDB 8T3L)	LPAx3 (EMDB-41006) (PDB 8T3M)	LPAx4 (EMDB-40940) (PDB 8T0C)
**Data collection and processing**						
Magnification	105,000×	105,000×	105,000×	105,000×	105,000×	105,000×
Voltage (kV)	300	300	300	300	300	300
Electron exposure (e^−^ Å^−^^2^)	45.8	45.8	45.8	45.8	45.8	45.8
Defocus range (μm)	0.5–2.0	0.5–2.0	0.5–2.0	0.5–2.0	0.5–2.0	0.5–2.0
Pixel size (Å)	0.835	0.835	0.835	0.835	0.835	0.835
Symmetry imposed	C4	C1	C2	C1	C1	C4
Initial particle images (no.)	240,263	240,263	240,263	240,263	240,263	240,263
Final particle images (no.)	4,090	7,921	2,030	7,916	10,602	11,119
Map resolution (Å)	3.3	3.5	3.7	3.6	3.5	3.5
FSC threshold	0.143	0.143	0.143	0.143	0.143	0.143
Map resolution range (Å)	3.0–19	3.4–6.8	3.7–8.6	3.6–8.0	3.5–7.8	2.8–11.9
**Refinement**						
Initial model used (PDB code)	7L2P	7L2P	7L2P	7MZD	7MZD	7MZD
Map sharpening *B* factor (Å^2^)	NA	−76.3	−79.9	−75.1	−90.1	NA
Model composition						
Nonhydrogen atoms	17,433	10,609	10,082	10,066	11,156	17,398
Protein residues	2,124	1,273	1,198	1,197	1,345	2,128
Ligands	5	5	5	6	6	6
*B* factors (min/max/mean Å^2^)						
Protein	64.90/177.90/120.66	42.25/97.04/62.10	30.98/75.19/43.98	30.24/87.68/50.64	44.17/103.68/64.26	47.13/161.23/99.92
Ligand	70.00/91.19/91.00	41.82/60.80/57.59	32.54/40.27/39.83	23.02/51.00/46.32	36.62/60.92/58.30	35.05/67.49/66.40
Root mean square deviations						
Bond lengths (Å)	0.37	0.32	0.46	0.32	0.34	0.28
Bond angles (°)	0.55	0.48	0.60	0.49	0.50	0.48
**Validation**						
MolProbity score	2	1	2	1	1	1
Clashscore	5	4	4	5	7	5
Poor rotamers (%)	2	1	3	1	1	0
Ramachandran plot						
Favored (%)	90	93	94	93	92	94
Allowed (%)	10	7	6	7	8	6
Disallowed (%)	0	0	0	0	0	0

NA, not applicable.

#### Empty-pocket TRPV1 at 4 °C (grid 1)

Empty-pocket TRPV1-nanodisc (described above) was kept on ice before applying to grids in a Vitrobot kept at 4 °C during grid preparation. Data were collected at S^2^C^2^ using TEM Gamma.

#### Empty-pocket TRPV1 at 25 °C (grid 2)

Empty-pocket TRPV1-nanodisc was kept on ice and briefly warmed to room temperature for 5 min before applying to grids in a Vitrobot equilibrated at 25 °C. Data were collected at S^2^C^2^ using TEM Beta.

#### PI–Br_4_ (grid 3) and PIP_2_–Br_4_ (grid 4)

TRPV1-nanodisc containing brominated phosphoinositide was kept on ice and applied to grids using a Vitrobot equilibrated at 25 °C. Data were collected at UCSF.

#### DiC8-PIP_2_ (grid 5)

DiC8-PIP_2_ was dissolved in buffer containing 20 mM HEPES (pH 7.5), 150 mM NaCl and 0.1 mM TCEP·HCl to a stock concentration of 1 mM. DiC8-PIP_2_ was applied to TRPV1-nanodiscs to a final concentration of 50 μM for 30 min on ice before applying sample to grids using a Vitrobot equilibrated at 15 °C. Data were collected at UCSF.

#### LPA (grid 6)

LPA was dissolved in DMSO to a working stock of 5 mM. LPA was applied to a final concentration of 50 μM (1% vehicle) to TRPV1-nanodiscs kept at room temperature for 30 min. Sample was applied to grids using a Vitrobot equilibrated at 25 °C. Data were collected at UCSF.

### Image processing

Cryo-EM data processing is illustrated in Supplementary Figs. 1–7, and derived Fourier shell correlation (dFSC) curves, angular distribution plots and local resolution are provided in Supplementary Fig. 8. In general, motion correction on movie stacks was processed on-the-fly using MotionCorr2 (ref. ^23^) in Scipion and binned 2 × 2 with Fourier cropping to 0.835 Å per pixel (UCSF Krios) or to 0.68 Å per pixel (S^2^C^2^). Dose-weighted micrographs were visually inspected to remove bad micrographs before further processing by cryoSPARC^24^. Patch-based contrast transfer function (CTF) estimation was performed in cryoSPARC. Micrographs with estimated resolution poorer than 4.5 Å were discarded. EMD-8118 (apo TRPV1 in nanodisc) was used to make 25 templates for template picking. Picks were extracted and binned 4 × 4 by Fourier cropping and reference-free two-dimensional (2D) classification was used to remove non-TRPV1-nanodisc picks. Extracted particles were then subjected to reference-based 3D classification (ref. EMD-8118, low-pass filtered 12 Å) RELION^25^ to remove low-resolution and featureless particles. Further processing for each dataset continued as stated below. Resolutions were determined according to the gold-standard Fourier shell correlation of 0.143 criterion^26^.

#### Empty-pocket TRPV1 4 °C

Particles were enriched for high-resolution features using symmetry expansion followed by focused classification as previously described^27^. Specifically, particles were refined using 3D Auto Refine with C4 symmetry in RELION using EMD-8118 as a reference (low-pass filter 12 Å). These refined particles were symmetry-expanded using C4 symmetry, and a mask focused on the VBP was used for background subtraction, followed by 3D classification on the symmetry-expanded particles. Classification parameters: ref. EMD-8118 (no low-pass filter), regularization parameter *T* = 80, symmetry C1, no image alignment. Particles without defined vanilloid pocket features were discarded. Particles containing monomers with four distinguishable pockets were than taken to cryoSPARC for nonuniform refinement (C4 symmetry) and then sharpened in PHENIX^28^ using half-map sharpening. Final resolution was 2.9 Å.

#### Empty-pocket TRPV1 25 °C

Selected particles from 3D classification were refined in cryoSPARC using nonuniform refinement and C4 symmetry. Final resolution was 3.7 Å.

#### PI–Br_4_

Selected particles from 2D classification were refined in RELION using C4 symmetry (reference EMD-8118, low-pass filtered 12 Å) to 2.5 Å. Particles were then 3D classified in RELION using no image alignment, and selected 3D classes with well-defined lipid features were refined in RELION as before. To further clarify bromine features, particles were subjected to the symmetry expansion and focused classification regiment as stated for empty-pocket TRPV1 at 4 °C. Classification parameters were regularization parameter *T* = 80, no low-pass on reference and no image alignment. Classes with all four monomers containing well-resolved phosphoinositide densities were refined in cryoSPARC using nonuniform refinement and C4 symmetry. The consensus map reached 2.2 Å resolution.

The consensus map showed a density projecting from the main-chain acyl density indicative of a secondary conformation for the lipid tail. Phosphoinositide tail conformations were further resolved with another round of refinement in RELION and symmetry expansion and focused classification (regularization parameter *T* = 80, no low-pass on reference, no image alignment). Two classes emerged with distinct tail conformations, named conformation 1 (tail projecting upward in the binding pocket as seen in other structures) and conformation 2 (tail projecting toward the membrane). Conformation 1 particles were combined, and conformation 2 particles were combined, and the subtracted particles (focused on the vanilloid pocket) were refined in RELION using EMD-8118 as the reference and a low pass filter of 12 Å and sharpened using Post Process in RELION. The final resolution reached 2.3 Å for both conformations. Conformation 1 is used in the main text figures as a representative of PI–Br_4_.

#### PIP_2_–Br_4_

Particles selected from 2D classes were extracted and then refined using cryoSPARC reference-based (EMD-8118) nonuniform refinement. As the sample is very homogeneous, 3D classification did not improve data quality and so was not used in the final data. The map was resolved to 2.4 Å.

#### DiC8-PIP_2_

Particles were refined in RELION using EMD-8118 as a reference low-pass filtered to 12 Å. Particles were then subjected to the symmetry expansion and focused classification regiment as stated above (regularization parameter *T* = 40, reference low-pass filtered to 12 Å, no image alignment). Low-resolution particles were excluded and two conformations of diC8-PIP_2_ emerged, a closed conformation and a dilated-state conformation. Particles containing all four monomers containing either diC8-PIP_2_ in the closed state or in the dilated state were taken further for refinement in RELION (reference EMD-8118, low-pass filtered 12 Å) and then sharpened using Post Process in RELION. The final resolution for closed was 3.0 Å and for dilated 3.6 Å.

#### LPA

The consensus map for LPA resolved to 3.0 Å. To further sperate LPA-bound from PI-bound subunits, all particles in the final dataset were subjected to symmetry expansion followed by focused classification as stated above. Classification parameters were ref. EMD-8118 (no low-pass filter), regularization parameter *T* = 80, symmetry C1 and no image alignment. Classes were whitelisted for further processing based on clarity of the ligand features, and the ligand content (LPA or lipid) was visually assigned to each class. Particles were grouped on the basis of the number of LPA bound (0, 1, 2, 3 or 4) and the arrangement (neighboring versus opposite pockets) of how the ligand is bound in tetrameric particles. New star files were generated for each group to calculate a 3D reconstruction followed by further refinement.

Particles with either zero or four LPA molecules bound were refined in cryoSPARC using nonuniform refinement with C4 symmetry, followed by PHENIX^28^ half-map sharpening. For other ligand-bound particles, transmembrane-region-focused and local refinement was performed on the prealigned particles in RELION. The resulting maps were sharpened using RELION Post-Process and the transmembrane mask was used as the solvent mask. Reconstructions with LPA bound in one, two neighboring or three subunits were refined without symmetry. For reconstruction with two LPA bound in two opposite subunits, particles were first subjected to 3D classification using one class, regularization parameter *T* = 40, symmetry C2, transmembrane mask and local searches to obtain the initial angles. The star file and resulting map were used in 3D Auto Refine as the input star file and the reference (low-pass filtered to the classification resolution of 4.1 Å), respectively, and refinement was carried out using a transmembrane mask and local searches. The resulting map was sharpened using RELION Post-Process and the transmembrane mask as the solvent mask.

### Model building

Side and top views of the models fit into their respective maps are shown in Supplementary Fig. 9. Resting TRPV1 (PDB-7L2P) or TRPV1-RTX (PDB-7MZD) was used as the starting model and docked into the sharpened maps using UCSF Chimera^29^, followed by manual adjustment based on the resolvable features of the maps. PDB and molecular restraint files for ligands were generated in PHENIX using eLBOW, and then manually docked into the ligand densities. For the general ‘resident lipid’, di-palmitoyl phosphatidylinositol was used as the starting structure and then the tails were shortened to fit the resolvable density. Models were refined using several iterations of PHENIX Real Space Refine and manual adjustments in COOT^30^. The quality of the refined models was determined using the wwPDB validation server^31^, and the results are presented in Tables 1 and 2.

#### Difference map analysis

Difference map analysis was used to emphasize densities for bromine atoms and phosphate groups in the PI–Br_4_ (conformation 1) and PIP_2_–Br_4_ maps. The final PDB models were used as the basis to generate calculated maps using the MolMap function in Chimera^29^ at 2.3 Å resolution for PI–Br_4_ and 2.4 Å for PIP_2_–Br_4_ (Extended Data Fig. 3). As resolution and *B* factors vary throughout the lipid densities, analyses were performed on a focused area around the bromine atoms (PI–Br_4_ and PIP_2_–Br_4_) or the inositol headgroup (for PIP_2_–Br_4_). For PI–Br_4_, the calculated map was generated from a PDB file that contained carbons 8–11 of the acyl tail and lacking the bromine atoms. For PIP_2_–Br_4_, a lipidic density comes near to bromine atoms of PIP_2_–Br_4_ and forms a connecting density. Therefore, the analysis PDB contained carbons 8–11 of the PIP_2_–Br_4_ acyl tail (lacking bromine) and the immediate carbon density of the other lipid. The corresponding densities in the real maps were then isolated, and the TEMPy:DiffMap function in the CCPEM^32,33^ suite was used to generate difference maps between the real densities and the calculated densities.

### Pore radius determination, tunnel analysis, electrostatics calculation and structural figures

The pore radii were determined using the HOLE program^34^ and plotted in Excel. Tunnels within TRPV1 were identified, visualized and made into a figure using the program CAVER^35^. Surface electrostatic charge determination was performed, visualized and made into a figure in PyMOL. All other structural figures were made using UCSF ChimeraX^36,37^ and Adobe Illustrator.

### TRPV1 proteoliposome preparation

Defined-lipid liposomes were prepared as reported previously^7^. Minimal functional rat TRPV1 with an N-terminal 8xHis-MBP tag was expressed in Expi-293F cells for 2 days using the Expifectamine-293 Transfection Kit (Thermo Fisher Scientific). Transfected cells were then collected by centrifugation at 3,000*g* for 10 min at 4 °C, with the supernatant decanted and cell pellets flash-frozen in liquid nitrogen and stored at −80 °C until use. To purify TRPV1, pellets were thawed and resuspended with buffer containing 200 mM NaCl, 50 mM HEPES pH 8, 2 mM TCEP, 10% glycerol and protease inhibitors (Pierce tablet). Twenty millimolar DDM (Anatrace) was added to extract TRPV1, while incubating on a rotator for 2 h at 4 °C. Samples were spun at 20,000*g* for 1 h at 4 °C, with the supernatant being collected, filtered at 0.2 µm and combined with ~1 ml amylose resin (New England BioLabs) for at least 1 h of affinity binding. Beads were poured over a Poly-Prep column (Bio-Rad) and washed with ~20 ml purification buffer (containing 200 mM NaCl, 50 mM HEPES pH 8, 2 mM TCEP, 10% glycerol, 1 mM DDM and 10 µg ml^−1^ defined lipid mixture) to remove impurities. TRPV1 was eluted with purification buffer plus 20 mM maltose.

Additional defined lipid mixture was dried down under nitrogen gas and stored in a vacuum desiccator one day before the liposome prep. The dried lipid was dissolved in buffer containing 200 mM NaCl, 5 mM MOPS pH 7 and 2 mM TCEP, to achieve a final concentration of 5 mg ml^−1^. The lipid stock was left to sit for 30 min, sonicated for 10 min and subjected to ten freeze–thaw cycles with liquid nitrogen and hot water to ensure lipid dispersion. Lipids were further destabilized by addition of 4 mM DDM and left to rotate for 30 min at room temperature. The resulting lipid–detergent stock was combined with eluted TRPV1 to achieve the desired 1:5 protein-to-lipid mass ratio and left to equilibrate for 1 h at room temperature on a rotator. Bio-Bead SM-2 resin was then added in four doses (60 mg, 60 mg, 150 mg, 300 mg per 10 mg lipid sample) with 1 h room-temperature rotator incubations in-between. After the last Bio-Bead incubation, the mixture was left overnight (over 15 h) and transferred to a 4 °C rotator. The next day, Bio-Beads were removed by a Poly-Prep column (Bio-Rad) and washed with a small volume of minimal buffer (containing 200 mM NaCl and 5 mM MOPS pH 7). Liposomes were pelleted at 100,000*g* for 1 h at 4 °C. Liposome pellets were resuspended in 200 µl of minimal buffer, divided into 13 µl aliquots, flash-frozen in liquid nitrogen and stored at −80 °C until use.

The composition of defined lipids varied according to the experiment. Experiments involving DiC8-PIP_2_ used as defined lipids (by mol%) 55.2% DOPC, 36.8% POPG and 8% cholesterol consistent with the nanodisc reconstitution experiments. Experiments involving PIP_2_–Br_4_ used as defined lipids (by mol%) 48.2% POPE, 22.8% POPC, 22.5% POPG, 4.5% cholesterol and 2.1% sphingomyelin, to stay consistent prior literature^7^, either alone, supplemented with 4% PIP_2_ or supplemented with 4% PIP_2_–Br_4_ as required.

### Liposome electrophysiology

Liposome electrophysiology was performed as previously described^7^. The day before a patch clamp session, one aliquot of frozen liposomes was thawed, supplemented with an equal volume of minimal buffer (containing 200 mM NaCl and 5 mM MOPS pH 7) plus 40 mM sucrose, plated on a glass coverslip and dehydrated in a vacuum desiccator for at least 50 min. One-hundred microliters of minimal buffer were added to the dried liposomes to rehydrate them overnight. Before patching, 5 µl of rehydrated liposomes were pipetted onto additional coverslips with 100 µl of minimal buffer on them and left still for 2 h to allow liposomes to adhere to the glass.

Bathing solution was delivered by gravity perfusion and contained 140 mM NaCl, 5 mM KCl, 20 mM HEPES pH 7.6 and 2 mM MgCl_2_. Internal solution was kept identical to bathing solution. Pipette tips were pulled from BF150-86-10 borosilicate capillaries (Sutter Instruments) using a P-97 micropipette puller (Sutter Instruments) and fire-polished using a MF-830 microforge (Narishige), keeping tip resistances between 3 and 10 MΩ. Coverslips with liposomes were transferred to an IX71 inverted microscope setup (Olympus). The electrode tip was pressed up against a liposome using an MP-285 micromanipulator (Sutter Instruments), and pressure was applied orally until a giga-Ohm seal was achieved. The tip was then slowly retracted until just a patch of membrane was retained, accessing the inside-out patch configuration. The patch was then placed in front of the SmartSquirt microperfusion system (AutoMate Scientific) to apply chemical ligands. Voltage steps between −120 mV and +120 mV or, if needed, −200 mV and +200 mV were applied in 10 mV increments, and currents were recorded, via an AxoPatch 200B amplifier (Molecular Devices) and a Digidata 1550B digitizer (Molecular Devices). Signals were acquired at 20 kHz and filtered at 5 kHz. Data were analyzed post hoc in pClamp (Molecular Devices), Excel (Microsoft) and Python. For DiC8-PIP_2_ experiments, TRPV1-specific currents were calculated by subtracting the leak of the patch, estimated as the lowest magnitude peak in an all-point histogram of current amplitude in a given potential, from the mean current measured over the given potential. For PIP_2_–Br_4_ experiments, the leak could not be subtracted in the same way.

### Statistics and experimental design

Electrophysiological data are presented as mean ± standard error of the mean (s.e.m.) unless otherwise noted. Statistical testing was carried out in Python. The applicability of parametric tests was first assessed, examining whether the data followed assumptions about equal variance (Levene’s test) and normal distribution (Shapiro–Wilk test). Where either of these assumptions were violated, we used nonparametric tests, initially a Kruskal–Wallis one-way analysis of variance and post-hoc two-tailed Dunn’s test using Bonferroni correction. For all tests, a priori, we set *α* = 0.05 and represent statistical significance with the *P* value, as indicated in the figure legends. We selected sample sizes for all experiments based on our laboratory and others’ experience with similar assays.

### Reporting summary

Further information on research design is available in the Nature Portfolio Reporting Summary linked to this article.

## Online content

Any methods, additional references, Nature Portfolio reporting summaries, source data, extended data, supplementary information, acknowledgements, peer review information; details of author contributions and competing interests; and statements of data and code availability are available at 10.1038/s41594-024-01299-2.

## Supplementary information

Supplementary Information Supplementary Figs 1–8.

Reporting Summary

Supplementary Video 1 Comparing the closed and dilated states of TRPV1 bound with diC8-PIP_2_. Morphs between TRPV1 in the closed and dilated states were made using ChimeraX. Only the transmembrane regions of TRPV1 are shown for clarity.

Supplementary Video 2 Binding trajectory of LPA binding to TRPV1 in through the ‘opposite’ configuration. The binding of LPA to TRPV1 is shown for the trajectory of LPAx0 → LPAx1 → LPAx2 ‘opposite’ → LPAx3 → LPAx4. LPA binding is demonstrated as pink star bursts. Monomers are colored as red (A), yellow (B), green (C) and blue (D). Y511 and L574 are shown along with The S4–S5 linker, S5 helix and S6 helix. Morphs between the LPA structures were generated in ChimeraX.

Supplementary Video 3 Binding trajectory of LPA binding to TRPV1 in through the ‘neighbor’ configuration. The binding of LPA to TRPV1 is shown for the trajectory of LPAx0 → LPAx1 → LPAx2 ‘neighbor’ → LPAx3 → LPAx4. LPA binding is demonstrated as pink star bursts. Monomers are colored as red (A), yellow (B), green (C) and blue (D). Y511 and L574 are shown along with The S4–S5 linker, S5 helix and S6 helix. Morphs between the LPA structures were generated in ChimeraX.

## Source data

Source Data Fig. 3h Data for bar graph.

Source Extended Data Fig. 2b Data for bar graph.

## Figures and Tables

**Extended Data Fig. 9:**
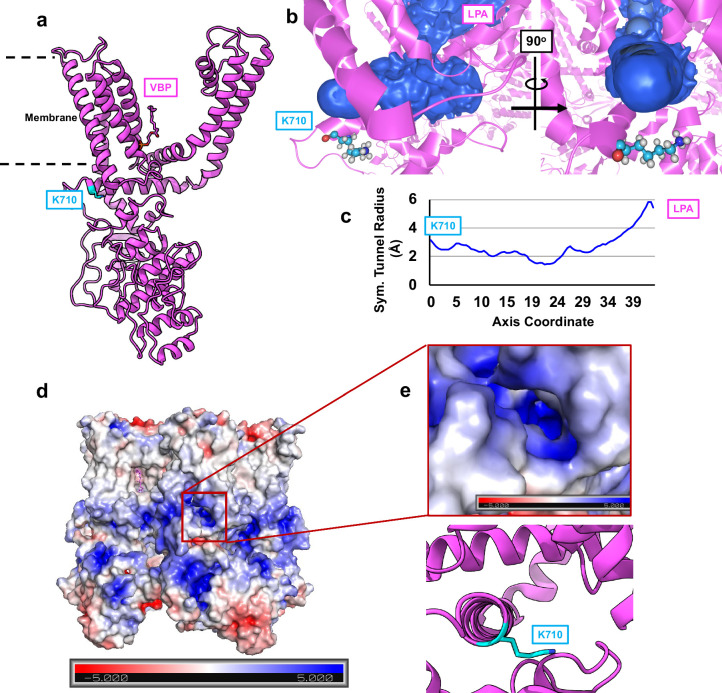
K710 marks a potential ingress tunnel for LPA. (**a**) K710 (blue) sits near the membrane surface and approximately 20 Å away from the VBP. (**b**) An access tunnel (blue) connecting K710 to the VBP as identified by the program CAVER. (**c**) Size of the tunnel as determined from CAVER. (**d**) Surface charge map of TRPV1 with location of putative tunnel indicated by red box. (**e**) Surface charge map of the tunnel identified by CAVER with the corresponding model of TRPV1-LPAx4 below.

## Data Availability

PDB structures and EMD maps be accessed by the following accession codes, respectively. Empty pocket 4 °C (8U3J, 41864), empty pocket 25 °C (8U3L, 41866), PI–Br_4_ consensus (8U4D, 41879), PI–Br_4_ conformation 1 (8U3A, 41855), PI–Br_4_ (8U3C, 41857), PIP_2_–Br_4_ (8U43, 41873, diC8-PIP_2_ closed (8U30, 41848), diC8-PIP_2_ dilated (8U2Z, 41847), LPAx0 (8T0E, 40941), LPAx1 (8T0Y, 40949), LPAx2 opposite (8T10, 40951), LPAx2 neighboring (8T3L, 41005), LPAx3 (8T3M, 41006) and LPAx4 (8T0C, 40940). Data for Fig. 3h and Extended Data Fig. 2b can be found in the provided source data file. Source data are provided with this paper.
