# Supplementary material for: Structural basis of TRPV1 modulation by endogenous bioactive lipids
Source: Nat Struct Mol Biol. 2024 May 2;31(9):1377–85. doi: 10.1038/s41594-024-01299-2 (PMC11402599; doi:10.1038/s41594-024-01299-2)
Supplement: Supplementary file 1 — Supplementary Figs 1–8. [file 41594_2024_1299_MOESM1_ESM.pdf]

nature structural & molecular biology

Article https://doi.org/10.1038/s41594-024-01299-2

Structural basis of TRPV1 modulation by endogenous bioactive lipids

In the format provided by the authors and unedited

Table of Contents

Figures

Supplementary Figure 1	2
Supplementary Figure 2	3
Supplementary Figure 3	4
Supplementary Figure 4	5
Supplementary Figure 5	6
Supplementary Figure 6	7
Supplementary Figure 7	8
Supplementary Figure 8	9
Supplementary Figure 9	10

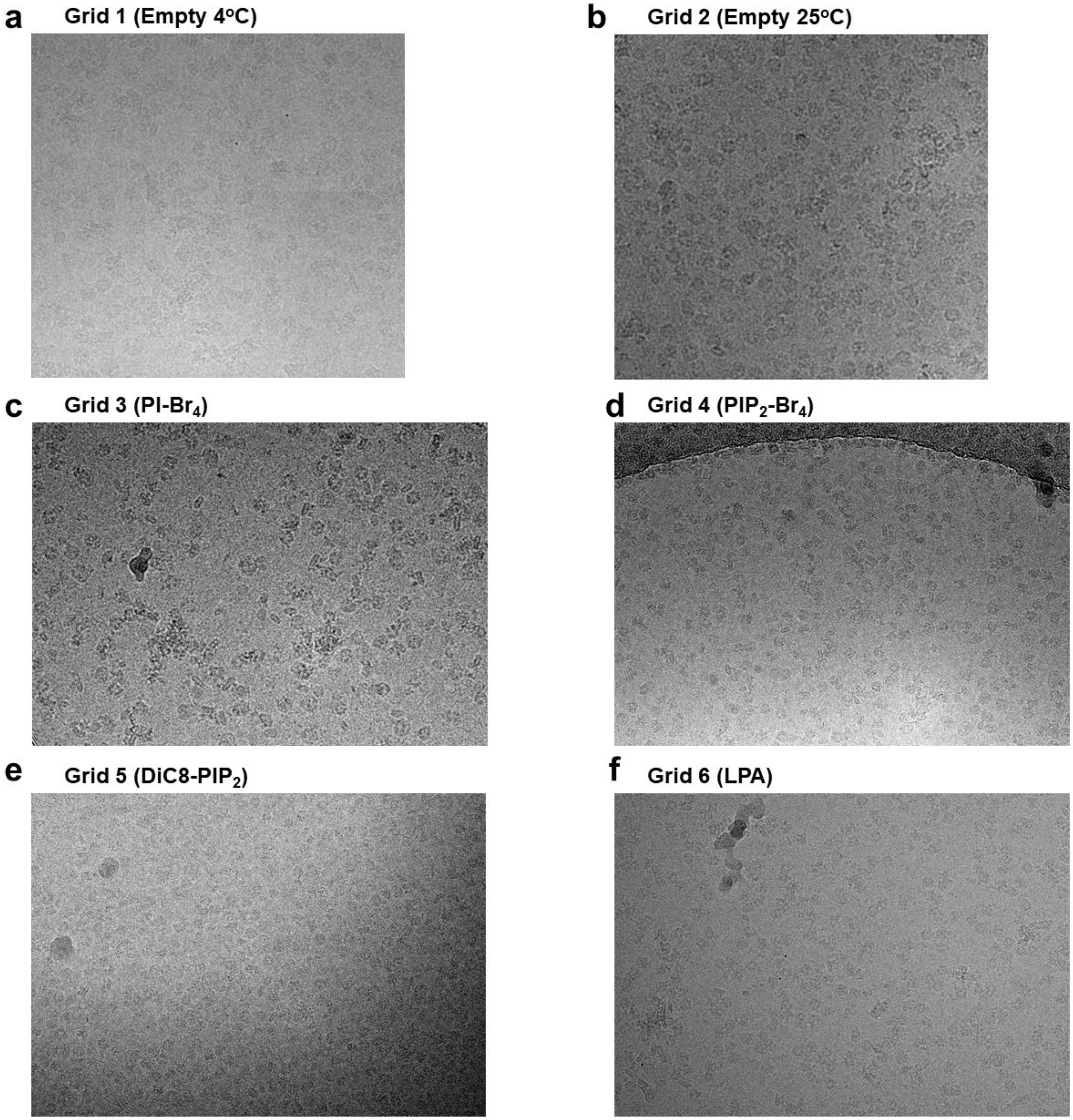

Supplementary Figure 1. Representative micrographs.

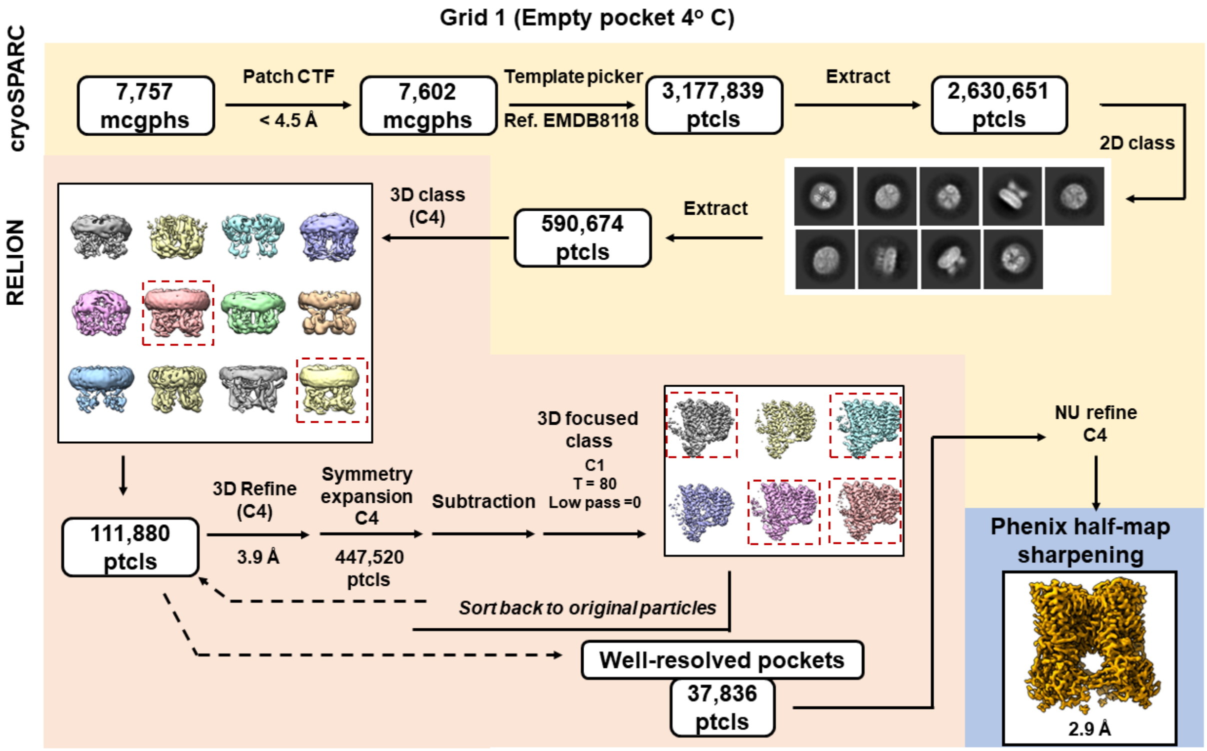

Supplementary Figure 2. Data processing scheme for Grid 1.

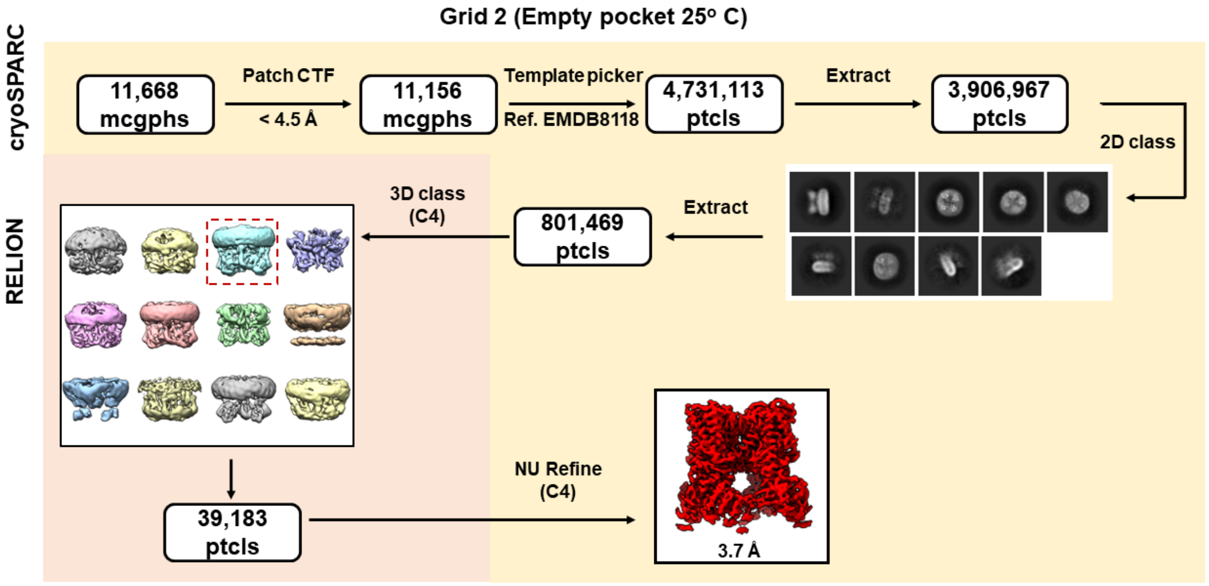

Supplementary Figure 3. Data processing scheme for Grid 2.

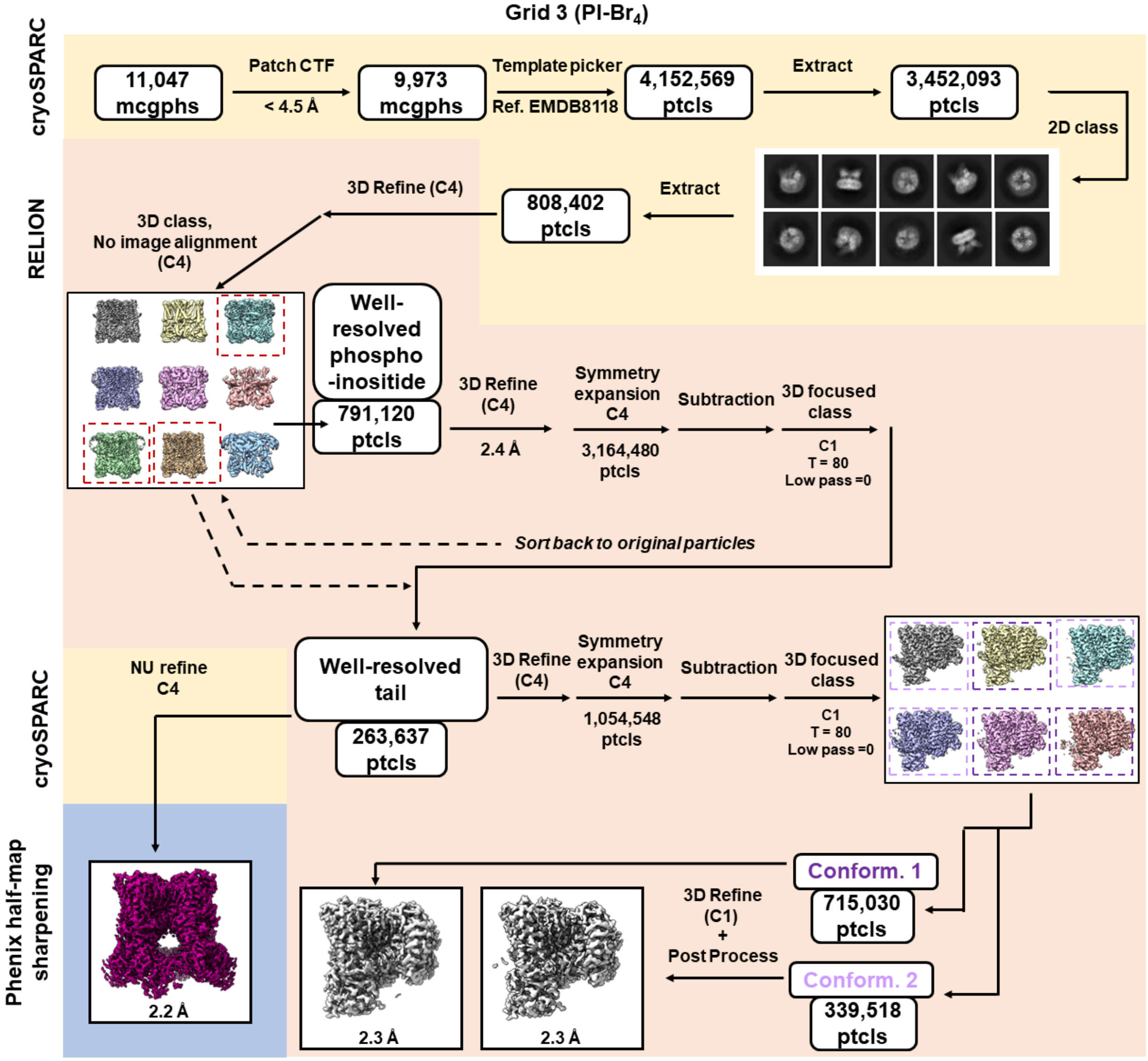

Supplementary Figure 4. Data processing scheme for Grid 3.

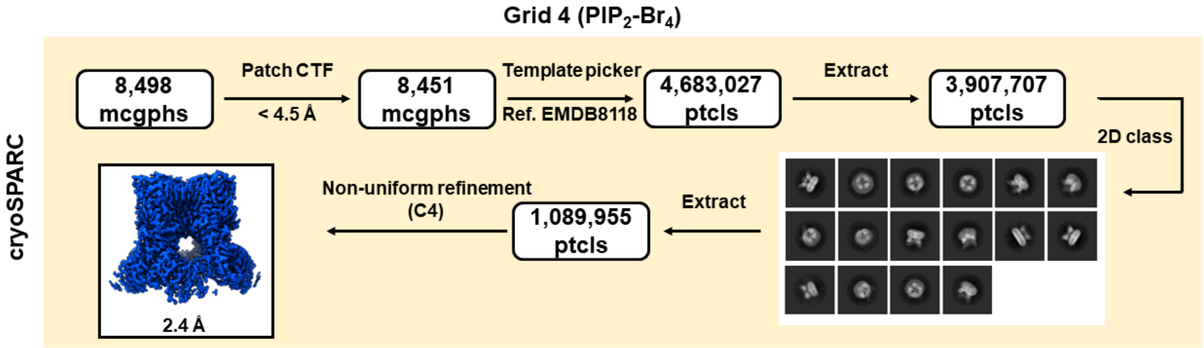

Supplementary Figure 5. Data processing scheme for Grid 4.

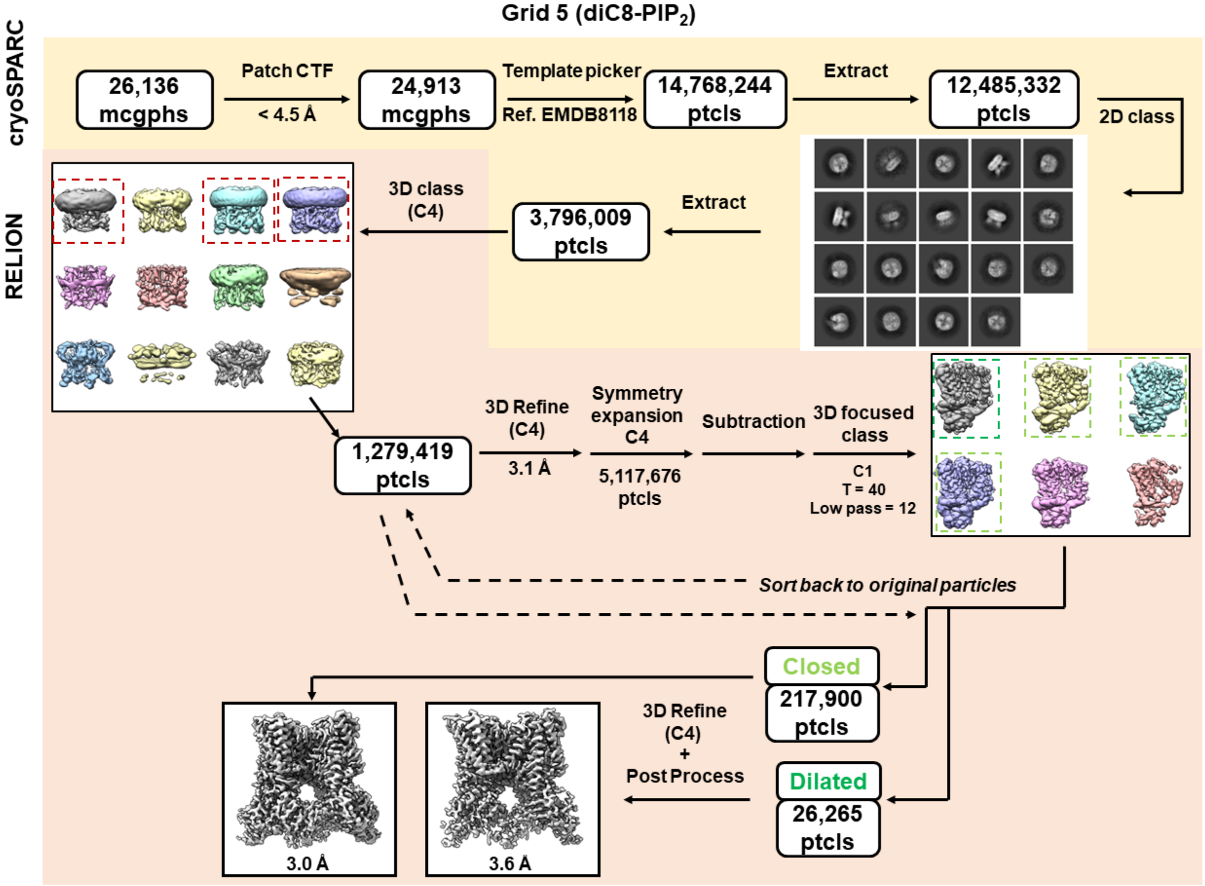

Supplementary Figure 6. Data processing scheme for Grid 5.

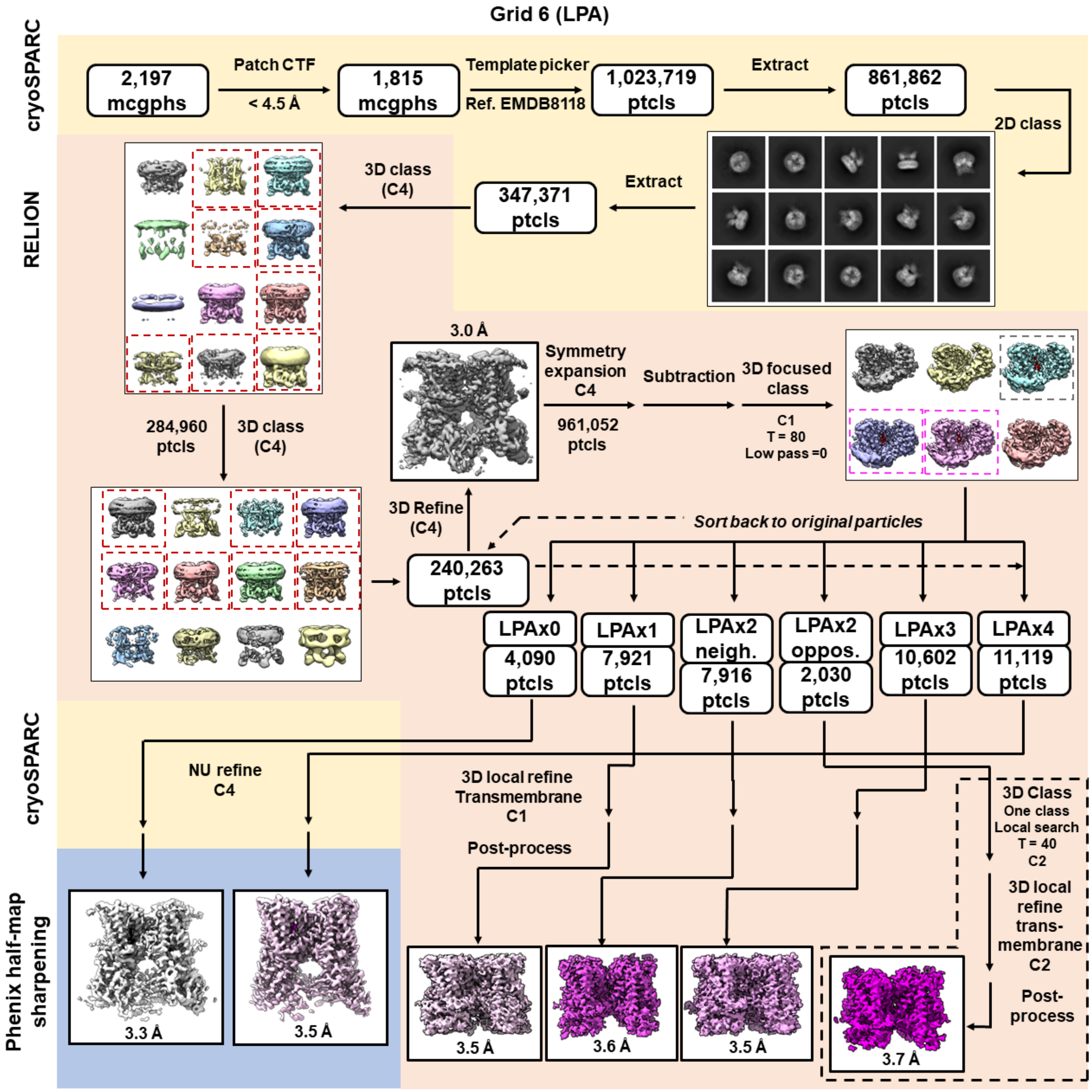

Supplementary Figure 7. Data processing scheme for Grid 6.

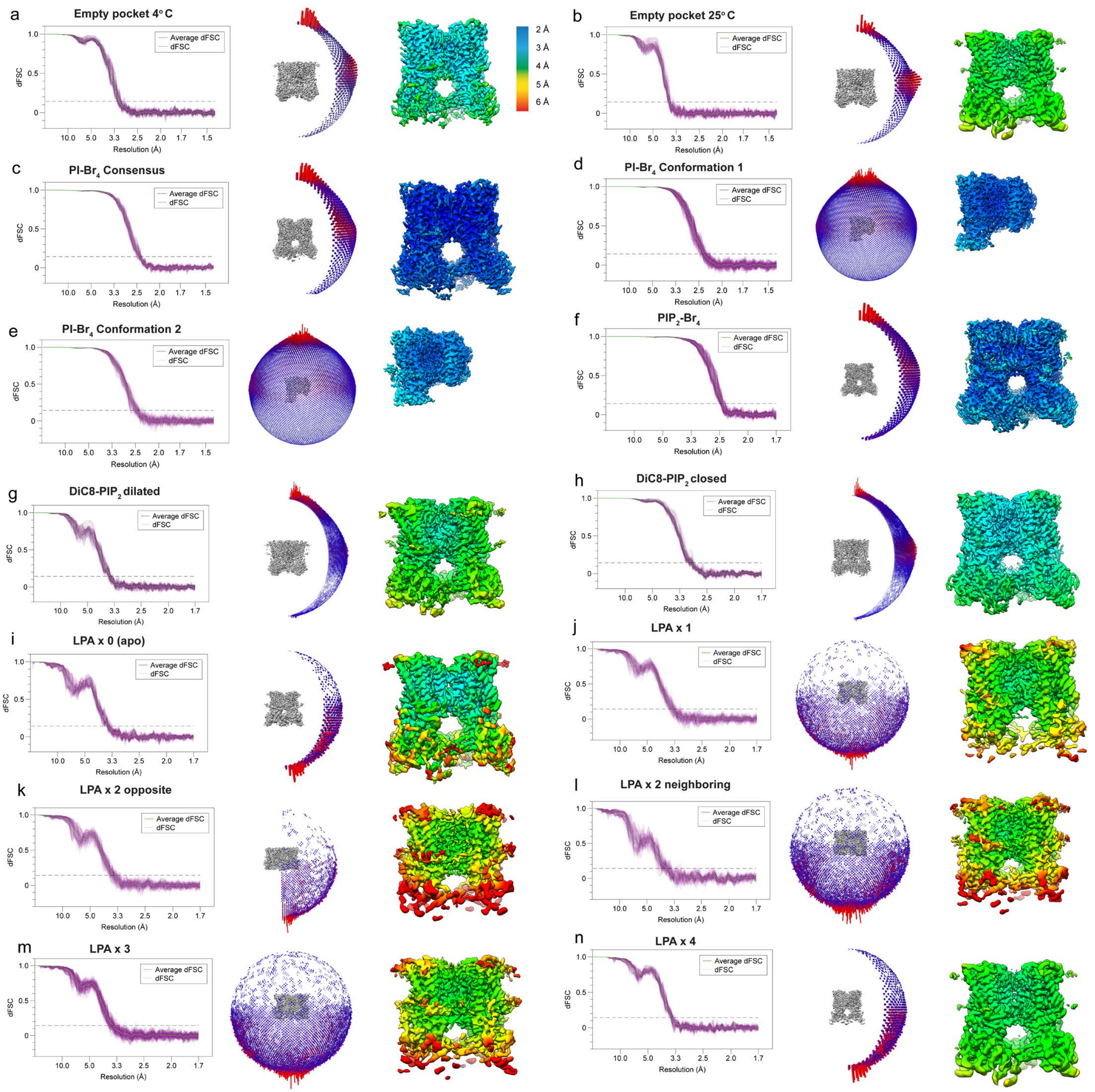

Supplementary Figure 8. (a-n) From left to right: plots of the dFSC curves, angular distribution plot, and local resolutions (colored according to key in (a)). For dFSC curves, gold standard FSC (0.143) is shown as a dashed line. Maps in d and e are from symmetry-expanded particles.

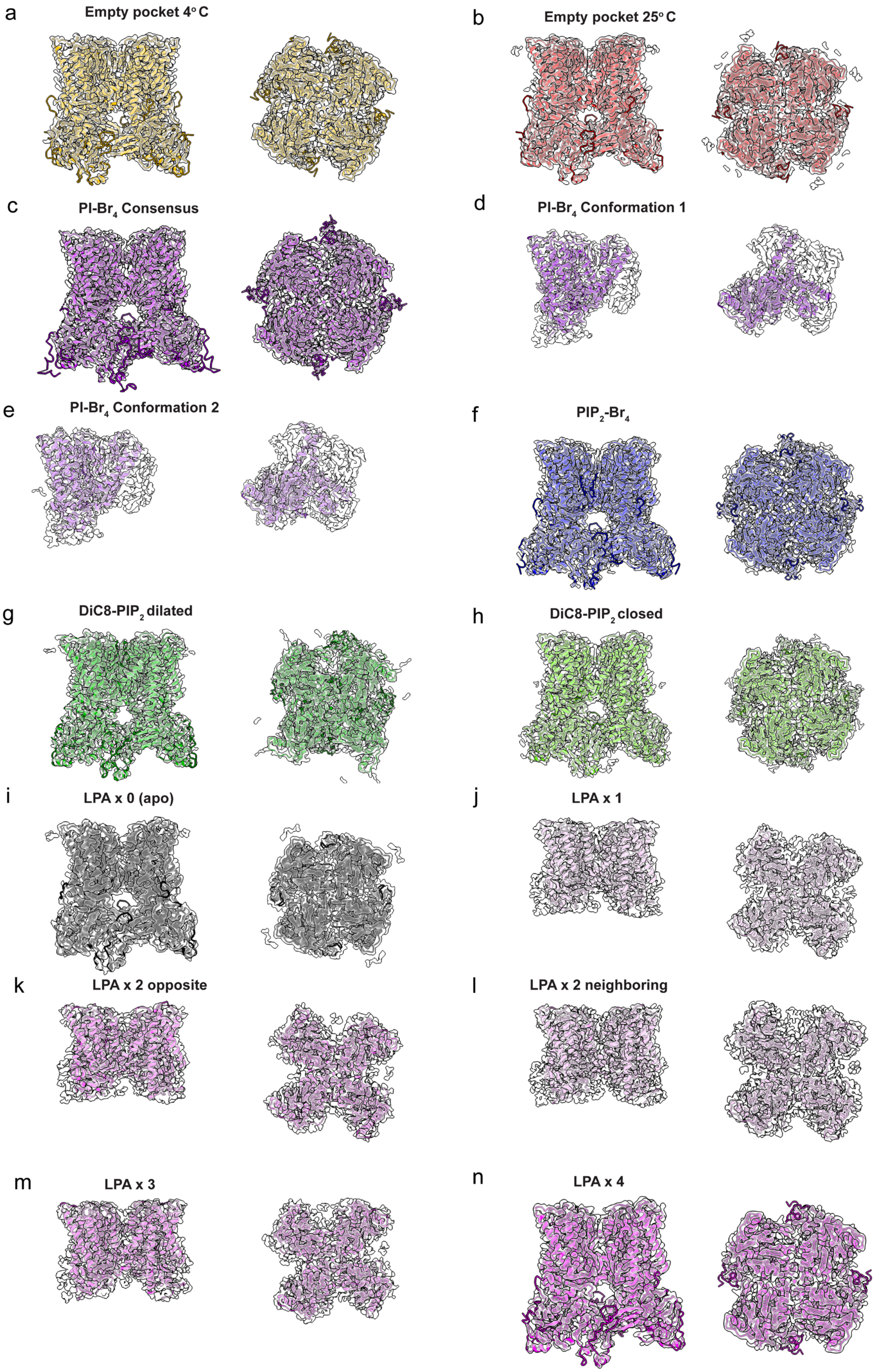

Supplementary Figure 9. (a-n) From left to right: Side and top views of all the models shown in their respective maps. Maps in d and e are from symmetry-expanded particles and models were built focusing on the binding site.
