# Supplementary material for: Structural basis of TRPV1 modulation by endogenous bioactive lipids
Source: Nat Struct Mol Biol. 2024 May 2;31(9):1377–85. doi: 10.1038/s41594-024-01299-2 (PMC11402599; doi:10.1038/s41594-024-01299-2)
Supplement: Supplementary file 2 — Reporting Summary [file 41594_2024_1299_MOESM2_ESM.pdf]

nature portfolio

Corresponding author(s): Yifan Cheng, David Julius

Last updated by author(s): Feb 19, 2023

Reporting Summary

Nature Portfolio wishes to improve the reproducibility of the work that we publish. This form provides structure for consistency and transparency in reporting. For further information on Nature Portfolio policies, see our Editorial Policies and the Editorial Policy Checklist.

Statistics

For all statistical analyses, confirm that the following items are present in the figure legend, table legend, main text, or Methods section.

n/a	Confirmed	
☐	☒	The exact sample size (*n*) for each experimental group/condition, given as a discrete number and unit of measurement
☐	☒	A statement on whether measurements were taken from distinct samples or whether the same sample was measured repeatedly
☐	☒	The statistical test(s) used AND whether they are one- or two-sided<br>*Only common tests should be described solely by name; describe more complex techniques in the Methods section.*
☒	☐	A description of all covariates tested
☐	☒	A description of any assumptions or corrections, such as tests of normality and adjustment for multiple comparisons
☐	☒	A full description of the statistical parameters including central tendency (e.g. means) or other basic estimates (e.g. regression coefficient) AND variation (e.g. standard deviation) or associated estimates of uncertainty (e.g. confidence intervals)
☐	☒	For null hypothesis testing, the test statistic (e.g. *F*, *t*, *r*) with confidence intervals, effect sizes, degrees of freedom and *P* value noted<br>*Give P values as exact values whenever suitable.*
☒	☐	For Bayesian analysis, information on the choice of priors and Markov chain Monte Carlo settings
☒	☐	For hierarchical and complex designs, identification of the appropriate level for tests and full reporting of outcomes
☒	☐	Estimates of effect sizes (e.g. Cohen's *d*, Pearson's *r*), indicating how they were calculated

Our web collection on statistics for biologists contains articles on many of the points above.

Software and code

Policy information about availability of computer code

Data collection	Serial EM 4, Scipion 3, Axon pClamp 10
Data analysis	cryoSPARC 4, RELION 4, Coot 0.8, PHENIX 1.2, Excel, HOLE 2, CAVER 3, wwPDB validate, PyMOL 2.5, Axon pClamp10, Python

For manuscripts utilizing custom algorithms or software that are central to the research but not yet described in published literature, software must be made available to editors and reviewers. We strongly encourage code deposition in a community repository (e.g. GitHub). See the Nature Portfolio guidelines for submitting code & software for further information.

Data

Policy information about availability of data

All manuscripts must include a data availability statement. This statement should provide the following information, where applicable:

- Accession codes, unique identifiers, or web links for publicly available datasets
- A description of any restrictions on data availability
- For clinical datasets or third party data, please ensure that the statement adheres to our policy

Models and maps are deposited in the Protein Data Base and Electron Microscopy Data Bank, respectively. Ascension codes are listed in the manuscript.

Research involving human participants, their data, or biological material

Policy information about studies with human participants or human data. See also policy information about sex, gender (identity/presentation), and sexual orientation and race, ethnicity and racism.

Reporting on sex and gender	No human research
Reporting on race, ethnicity, or other socially relevant groupings	No human research
Population characteristics	No human research
Recruitment	No human research
Ethics oversight	No human research

Note that full information on the approval of the study protocol must also be provided in the manuscript.

Field-specific reporting

Please select the one below that is the best fit for your research. If you are not sure, read the appropriate sections before making your selection.

☒ Life sciences ☐ Behavioural & social sciences ☐ Ecological, evolutionary & environmental sciences

For a reference copy of the document with all sections, see nature.com/documents/nr-reporting-summary-flat.pdf

Life sciences study design

All studies must disclose on these points even when the disclosure is negative.

Sample size

Sample sizes were determined according to standards in the field. For CryoEM, enough particles were collected to achieve best resolution. For electrophysiology, enough recordings were collected to observe generalizable trend (n>=10), consistent with our laboratory and others' experience with similar assays. As precedence for sample numbers, please consult: Caterina et al, 1997, Nature; Zhao et al, 2020, Nature; Nieto-Posadas et al, 2011, Nature Chemical Biology.

Data exclusions

Data were excluded as outlined in the text following standard procedures of the field. Data were excluded based on resolvability of the data and clarity of the EM densitities.

Replication

Grids were collected on once for the study as is the standard for the field because you can only collect on them once. For the electrophysiology, there were four main conditions probed - (1) repeated agonist/antagonist exposure in Data Figure 3h, and single agonist exposure in Extended Data Figure 2b for no phosphoinositide (2), PIP2 (3), and Br4-PIP2 (4) supplemented liposomes. For each of these conditions, recordings were repeated multiple times in a recording day, either minutes to hours apart. Data in condition 1 was collected in a sinle recording day, but each of conditions 2, 3, and 4 were done over at least two recording days. The data were similar within a day and between days. The data plotted is average of all those attempts

Randomization

Randomization is not needed

Blinding

Blinding not relevant for study because data processing requires knowing what the experimental conditions were to process the data and interpret results

Reporting for specific materials, systems and methods

We require information from authors about some types of materials, experimental systems and methods used in many studies. Here, indicate whether each material, system or method listed is relevant to your study. If you are not sure if a list item applies to your research, read the appropriate section before selecting a response.

Materials & experimental systems

n/a	Involved in the study	
☒	☐	Antibodies
☐	☒	Eukaryotic cell lines
☐	☐	Palaeontology and archaeology
☐	☐	Animals and other organisms
☐	☐	Clinical data
☒	☐	Dual use research of concern
☒	☐	Plants

Methods

n/a	Involved in the study	
☒	☐	ChIP-seq
☒	☐	Flow cytometry
☒	☐	MRI-based neuroimaging

Eukaryotic cell lines

Policy information about cell lines and Sex and Gender in Research

Cell line source(s)	HEK293 GnTi (minus), HEK expi293, obtained from Fisher
Authentication	Cell lines were not authenticated
Mycoplasma contamination	Mycoplasma contamination not performed. Cells were used for recombinant protein expression and data do not involve cell physiology.
Commonly misidentified lines (See ICLAC register)	HEK cells were used for recombinant protein expression. Cell physiology not accessed.
